# Surveillance for Violent Deaths — National Violent Death Reporting System, 48 States, the District of Columbia, and Puerto Rico, 2021

**DOI:** 10.15585/mmwr.ss7305a1

**Published:** 2024-07-11

**Authors:** Brenda L. Nguyen, Bridget H. Lyons, Kaitlin Forsberg, Rebecca F. Wilson, Grace S. Liu, Carter J. Betz, Janet M. Blair

**Affiliations:** 1 Division of Violence Prevention, National Center for Injury Prevention and Control, CDC

## Abstract

**Problem/Condition:**

In 2021, approximately 75,000 persons died of violence-related injuries in the United States. This report summarizes data from CDC’s National Violent Death Reporting System (NVDRS) on violent deaths that occurred in 48 states, the District of Columbia, and Puerto Rico in 2021. Results are reported by sex, age group, race and ethnicity, method of injury, type of location where the injury occurred, circumstances of injury, and other selected characteristics. This report introduces additional incident and circumstance variables, which now include child victim–specific circumstance information. This report also incorporates new U.S. Census Bureau race and ethnicity categories, which now account for more than one race and Native Hawaiian or other Pacific Islander categories and include updated denominators to calculate rates for these populations.

**Period Covered:**

2021.

**Description of System:**

NVDRS collects data regarding violent deaths from death certificates, coroner and medical examiner records, and law enforcement reports. This report includes data collected for violent deaths that occurred in 2021. Data were collected from 48 states (all states with exception of Florida and Hawaii), the District of Columbia, and Puerto Rico. Forty-six states had statewide data, two additional states had data from counties representing a subset of their population (31 California counties, representing 64% of its population, and 13 Texas counties, representing 63% of its population), and the District of Columbia and Puerto Rico had jurisdiction-wide data. NVDRS collates information for each violent death and links deaths that are related (e.g., multiple homicides, homicide followed by suicide, or multiple suicides) into a single incident.

**Results:**

For 2021, NVDRS collected information on 68,866 fatal incidents involving 70,688 deaths that occurred in 48 states (46 states collecting statewide data, 31 California counties, and 13 Texas counties), and the District of Columbia. The deaths captured in NVDRS accounted for 86.5% of all homicides, legal intervention deaths, suicides, unintentional firearm injury deaths, and deaths of undetermined intent in the United States in 2021. In addition, information was collected for 816 fatal incidents involving 880 deaths in Puerto Rico. Data for Puerto Rico were analyzed separately. Of the 70,688 deaths, the majority (58.2%) were suicides, followed by homicides (31.5%), deaths of undetermined intent that might be due to violence (8.2%), legal intervention deaths (1.3%) (i.e., deaths caused by law enforcement and other persons with legal authority to use deadly force acting in the line of duty, excluding legal executions), and unintentional firearm injury deaths (<1.0%). The term “legal intervention” is a classification incorporated into the *International Classification of Diseases, Tenth Revision*, and does not denote the lawfulness or legality of the circumstances surrounding a death caused by law enforcement.

Demographic patterns and circumstances varied by manner of death. The suicide rate was higher for males than for females. Across all age groups, the suicide rate was highest among adults aged ≥85 years. In addition, non-Hispanic American Indian or Alaska Native (AI/AN) persons had the highest suicide rates among all racial and ethnic groups. Among both males and females, the most common method of injury for suicide was a firearm. Among all suicide victims, when circumstances were known (84.4%), suicide was most often preceded by a mental health, intimate partner, or physical health problem or by a recent or impending crisis during the previous or upcoming 2 weeks. The homicide rate was higher for males than for females. Among all homicide victims, the homicide rate was highest among persons aged 20–24 years compared with other age groups. Non-Hispanic Black or African American (Black) males experienced the highest homicide rate of any racial or ethnic group. Among all homicide victims, the most common method of injury was a firearm. When the relationship between a homicide victim and a suspect was known, the suspect was most frequently an acquaintance or friend for male victims and a current or former intimate partner for female victims. Homicide most often was precipitated by an argument or conflict, occurred in conjunction with another crime, or, for female victims, was related to intimate partner violence. Nearly all victims of legal intervention deaths were male, and the legal intervention death rate was highest among men aged 30–34 years. The legal intervention death rate was highest among AI/AN males, followed by Black males. A firearm was used in the majority of legal intervention deaths. When circumstances were known, the most frequent circumstances reported for legal intervention deaths were as follows: the victim used a weapon in the incident and the victim had a substance use problem (other than alcohol use).

Other causes of death included unintentional firearm injury deaths and deaths of undetermined intent. Unintentional firearm injury deaths were most frequently experienced by males, non-Hispanic White (White) persons, and persons aged 15–24 years. These deaths most frequently occurred while the shooter was playing with a firearm and were precipitated by a person unintentionally pulling the trigger. The rate of deaths of undetermined intent was highest among males, particularly among AI/AN and Black males, and among adults aged 30–54 years. Poisoning was the most common method of injury in deaths of undetermined intent, and opioids were detected in nearly 80% of decedents tested for those substances.

**Interpretation:**

This report provides a detailed summary of data from NVDRS on violent deaths that occurred in 2021. The suicide rate was highest among AI/AN and White males, whereas the homicide rate was highest among Black males. Intimate partner violence precipitated a large proportion of homicides for females. Mental health problems, intimate partner problems, interpersonal conflicts, and acute life stressors were primary precipitating circumstances for multiple types of deaths examined.

**Public Health Action:**

Violence is preventable, and data can guide public health action. NVDRS data are used to monitor the occurrence of violence-related fatal injuries and assist public health authorities in developing, implementing, and evaluating programs, policies, and practices to reduce and prevent violent deaths. NVDRS data can be used to enhance prevention efforts into actionable strategies. States or jurisdictions have used their Violent Death Reporting System (VDRS) data to guide suicide prevention efforts and highlight where additional focus is needed. For example, North Carolina VDRS program data have played a significant role in expanding activities related to firearm safety and injury prevention. The program served as a primary data source for partners, which led to the creation of the Office of Violence Prevention in the state, focusing on combatting firearm-related deaths. In Maine, the VDRS provided data on law enforcement officer suicides that were used to help support a bill mandating mental health resiliency and awareness training in the state’s law enforcement training academy, along with plans for similar training addressing mental health, substance use, and alcohol problems among corrections officers. In addition, states and jurisdictions have also used their VDRS data to examine factors related to homicide in their state or jurisdiction. For example, Georgia VDRS collaborated with the City of Atlanta Mayor’s Office of Violence Reduction to develop two public dashboards that not only offer comprehensive data on violent deaths but also present data on the geographic distribution of populations disproportionately affected by violence to help inform violence prevention interventions.

## Introduction

According to National Vital Statistics System mortality data obtained from CDC’s Web-based Injury Statistics Query and Reporting System (WISQARS),* violence-related injuries led to 74,883 deaths in the United States in 2021 due to suicides, homicide, or legal intervention, with additional deaths due to unintentional firearm injuries (n = 549) and undetermined intent (n = 6,259)(*1*). Suicide was the 11th leading cause of death overall in the United States and disproportionately affected specific age and racial groups. By age group, suicide was the second leading cause of death for persons aged 10–34 years and was the fifth leading cause of death among adults aged 35–44 years. Non-Hispanic American Indian or Alaska Native (AI/AN) and non-Hispanic White (White) males had the highest rates of suicide compared with all other racial and ethnic groups and females. 

In 2021, homicide was the 16th leading cause of death overall in the United States but disproportionately affected young persons and non-Hispanic Black or African American (Black) males (*1*,*2*). Homicide was the third leading cause of death for children aged 1–14 years, the second leading cause of death for persons aged 15–24 years, and the third leading cause of death for persons aged 25–34 years. Homicide was the leading cause of death for Black males aged 15–24 years and the second leading cause of death for Black males aged 1–14 years (*2*).

Public health authorities require accurate, timely, and complete surveillance data to better understand and ultimately prevent the occurrence of violent deaths in the United States (*3*,*4*). In 2000, in response to an Institute of Medicine^†^ report noting the need for a national fatal intentional injury surveillance system (*5*), CDC began planning to implement the National Violent Death Reporting System (NVDRS) (*3*). The goals of NVDRS are to

- collect and analyze timely, high-quality data for monitoring the magnitude and characteristics of violent deaths at national, state, and local levels;
- ensure data are disseminated routinely and expeditiously to public health officials, law enforcement officials, policymakers, and the public;
- ensure data are used to develop, implement, and evaluate programs and strategies that are intended to reduce and prevent violent deaths and injuries at national, state, and local levels; and
- build and strengthen partnerships among organizations and communities at national, state, and local levels to ensure that data are collected and used to reduce and prevent violent deaths and injuries.

NVDRS is a state- and territory-based active surveillance system that collects data on the characteristics and circumstances associated with violence-related deaths among participating states, the District of Columbia, and Puerto Rico (*3*). Deaths collected by NVDRS include suicides, homicides, legal intervention deaths (i.e., deaths caused by law enforcement acting in the line of duty and other persons with legal authority to use deadly force, excluding legal executions), unintentional firearm injury deaths, and deaths of undetermined intent that might have occurred due to violence.^§^ The term “legal intervention” is a classification incorporated into the *International Classification of Diseases, Tenth Revision*, (ICD-10) (*6*) and does not denote the lawfulness or legality of the circumstances surrounding a death caused by law enforcement.

Before implementation of NVDRS, single data sources (e.g., death certificates) provided only limited information and few circumstances from which to understand patterns of violent deaths. NVDRS filled this surveillance gap by providing more detailed information. NVDRS is the first system to 1) provide detailed information on circumstances precipitating violent deaths, 2) link multiple source documents so that each incident can contribute to the study of patterns of violent deaths, and 3) link multiple deaths that are related to one another (e.g., multiple homicides, suicide pacts, or homicide followed by suicide of the suspect).

NVDRS data collection began in 2003 with six participating states (Maryland, Massachusetts, New Jersey, Oregon, South Carolina, and Virginia) and has gradually expanded (Figure). Since 2018, CDC has provided NVDRS funding to all 50 states, the District of Columbia, and Puerto Rico. NVDRS data are updated annually and are available to the public through WISQARS (https://wisqars.cdc.gov/nvdrs). Case-level NVDRS data are available to interested researchers who meet eligibility requirements via the NVDRS Restricted Access Database (https://www.cdc.gov/nvdrs/about/nvdrs-data-access.html).

**FIGURE:**
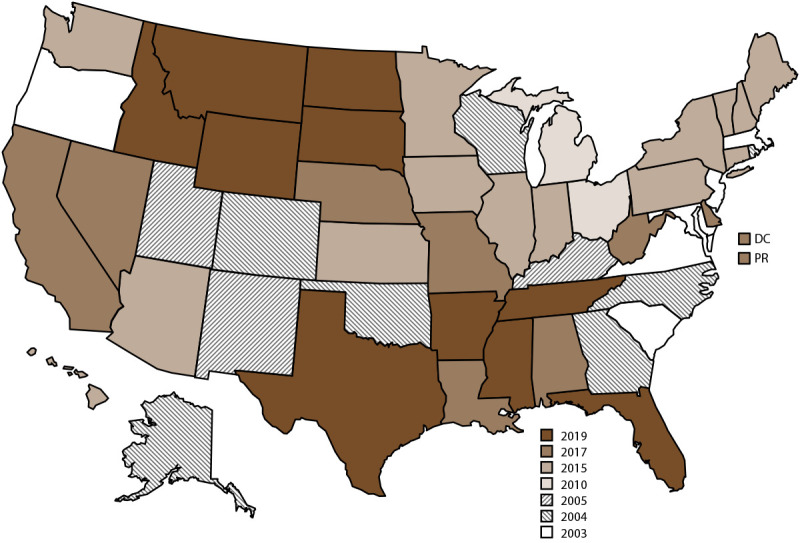
States and jurisdictions participating in the National Violent Death Reporting System, by year of initial data collection* — United States and Puerto Rico, 2003–2021 **Abbreviations:** DC = District of Columbia; NVDRS = National Violent Death Reporting System; PR = Puerto Rico. * Map of United States indicates the year in which the state or jurisdiction began collecting data in NVDRS. Beginning in 2019, all 50 U.S. states, the District of Columbia, and Puerto Rico were participating in the system. California began collecting data for a subset of violent deaths in 2005 but ended data collection in 2009; however, in 2017, California resumed collecting data for a subset of violent deaths and expanded coverage in subsequent years. In 2021, California collected data for violent deaths in 31 counties (Amador, Butte, Colusa, Fresno, Glenn, Humboldt, Imperial, Kings, Lake, Lassen, Los Angeles, Mendocino, Merced, Modoc, Mono, Orange, Placer, Sacramento, San Benito, San Diego, San Francisco, San Luis Obispo, San Mateo, Santa Cruz, Shasta, Siskiyou, Solano, Sonoma, Tehama, Ventura, and Yolo) representing 62% of the state’s population. Michigan collected data for a subset of violent deaths during 2010–2013 and expanded to collecting statewide data beginning in 2014. In 2016, Illinois, Pennsylvania, and Washington began collecting data on violent deaths in a subset of counties that represented at least 80% of all violent deaths in their state or in counties. Washington began collecting statewide data for all violent deaths beginning in 2018, and Illinois and Pennsylvania began collecting statewide data beginning in 2020. In 2019, Texas began collecting data for a subset of violent deaths and expanded coverage in subsequent years. In 2021, Texas collected data for violent deaths that occurred in 13 counties (Bell, Bexar, Collin, Dallas, Denton, El Paso, Fort Bend, Harris, Montgomery, Nueces, Tarrant, Travis, and Williamson) representing approximately 63% of the state’s population.

This report summarizes NVDRS data on violence-related deaths that occurred in 48 states, the District of Columbia, and Puerto Rico in 2021. Forty-six states collected statewide data (Alabama, Alaska, Arizona, Arkansas, Colorado, Connecticut, Delaware, Georgia, Idaho, Illinois, Indiana, Iowa, Kansas, Kentucky, Louisiana, Maine, Maryland, Massachusetts, Michigan, Minnesota, Mississippi, Missouri, Montana, Nebraska, Nevada, New Hampshire, New Jersey, New Mexico, New York, North Carolina, North Dakota, Ohio, Oklahoma, Oregon, Pennsylvania, Rhode Island, South Carolina, South Dakota, Tennessee, Utah, Vermont, Virginia, Washington, West Virginia, Wisconsin, and Wyoming). The two remaining states collected data from a subset of counties in their states (31 California counties^¶^ and 13 Texas counties**). Compared with the NVDRS report for 2020 (*7*), this report for 2021 includes additional incident and child victim–specific variables, and new U.S. Census Bureau race and ethnicity categories, which now account for more than one race and Native Hawaiian and other Pacific Islander (NH/PI) categories. Data for Florida and Hawaii were ineligible to be included in this report because the data did not meet the completeness threshold for circumstances (see Inclusion Criteria).

## Methods

NVDRS compiles information from three required data sources: death certificates, coroner and medical examiner records, and law enforcement reports (*3*). Certain participating Violent Death Reporting System (VDRS) programs might also collect information from secondary data sources (e.g., child fatality review team data, Federal Bureau of Investigation Supplementary Homicide Reports, or crime laboratory data). NVDRS combines information for each death and links deaths that are related (e.g., multiple homicides, homicide followed by suicide, or multiple suicides) into a single incident. The ability to analyze linked data can provide a more comprehensive understanding of violent deaths. Participating VDRS programs use vital statistics death certificate files or coroner or medical examiner records to identify deaths meeting the NVDRS case definition (see Manner of Death). Each VDRS program reports violent deaths of residents that occurred within the state, district, or territory (i.e., resident deaths) and those of nonresidents who experienced a fatal injury within the state, district, or territory (i.e., occurrent deaths). When a case definition matching a violent death is identified, NVDRS data abstractors link source documents, link deaths within each incident, code data elements, and write brief narratives of the incident.

In NVDRS, a violent death is defined as a death resulting from the intentional use of physical force or power, threatened or actual, against oneself, another person, or a group or community (*3*). NVDRS collects information about homicides, suicides, deaths by legal intervention (excluding legal executions), and deaths of undetermined intent that might have occurred due to violence. NVDRS also collects information on unintentional firearm injury deaths. The primary reason is to provide a more complete picture of firearm-related injury deaths in the U.S. (see Manner of Death). NVDRS cases are determined based on ICD-10 cause of death codes (*6*) or the manner of death assigned by a coroner, medical examiner, or law enforcement officer. Cases are included if they are assigned *International Classification of Diseases, Tenth Revision* (ICD-10) cause of death codes (Box 1) or a manner of death specified in at least one of the three primary data sources consistent with NVDRS case definitions.

BOX 1 ***International Classification of Diseases, Tenth Revision* codes used in the National Violent Death Reporting System, 2021**

**Table:** 

Manner of death	Death ≤1 year after injury	Death >1 year after injury	Death any time after injury
Intentional self-harm (suicide)	X60–X84	Y87.0	U03 (attributable to terrorism)
Assault (homicide)	X85–X99, Y00–Y09	Y87.1	U01, U02 (attributable to terrorism)
Event of undetermined intent	Y10–Y34	Y87.2, Y89.9	Not applicable
Unintentional exposure to inanimate mechanical forces (firearms)	W32–W34	Y86	Not applicable
Legal intervention (excluding executions, Y35.5)	Y35.0–Y35.4, Y35.6, Y35.7	Y89.0	Not applicable

NVDRS is an incident-based system, and all decedents associated with an incident are grouped in one record. Decisions about whether two or more deaths are related and belong to the same incident are made based on the timing of the injuries rather than on the timing of the deaths. Deaths resulting from injuries that are clearly linked by source documents and occur within 24 hours of each other are considered part of the same incident. Examples of an incident include 1) a single isolated violent death, 2) two or more related homicides (including legal intervention deaths) in which the fatal injuries were inflicted <24 hours apart, 3) two or more related suicides or deaths of undetermined intent in which the fatal injuries were inflicted <24 hours apart, and 4) a homicide followed by a suicide in which both fatal injuries were inflicted <24 hours apart (*8*).

Information collected from each data source is entered into the NVDRS web-based system (*3*). This system streamlines data abstraction by allowing abstractors to enter data from multiple sources into the same incident record. Internal validation checks, hover-over features that define selected fields, and other quality control measures are also included within the system. Primacy rules and hierarchal algorithms related to the source documents occur at the local VDRS program level. CDC provides access to the web-based system to each VDRS program. VDRS program personnel are provided ongoing training to learn and adhere to CDC guidance regarding the coding of all variables and technical assistance to help increase data quality. Information abstracted into the system is deidentified at the local VDRS program level, and data are transmitted continuously via the web to a CDC-based server. This activity was reviewed by CDC, deemed not research, and was conducted consistent with applicable Federal law and CDC policy.^^††^^

### Manner of Death

A manner (i.e., intent) of death for each decedent is assigned by a trained abstractor who integrates information from all source documents. The abstractor-assigned manner of death must be consistent with at least one required data source; typically, all source documents are consistent regarding the manner of death. When a discrepancy exists, the abstractor must assign a manner of death based on a preponderance of evidence in the source documents; however, such occurrences are rare (*8*). For example, if two sources report a death as a suicide and a third reports it as a death of undetermined intent, the death is coded as a suicide.

NVDRS data are categorized into five abstractor-assigned manners of death: 1) suicide, 2) homicide, 3) legal intervention death, 4) unintentional firearm injury death, and 5) death of undetermined intent. The case definitions for each manner of death are described as follows:

- **Suicide.** A suicide is a death among persons aged ≥10 years resulting from the use of force against oneself when a preponderance of evidence indicates that the use of force was intentional. Although suicide deaths for children aged 5–9 years are collected in NVDRS, they are not included in total numbers, nor are they included as part of age-specific numbers or rates because of the small number of suicide deaths per year in this age group. This category includes the following scenarios: 1) deaths of persons who intended only to injure themselves rather than die by suicide; 2) persons who initially intended to die by suicide and changed their minds but still died as a result of the act; 3) deaths associated with risk-taking behavior without clear intent to inflict a fatal self-injury but associated with high risk for death (e.g., participating in Russian roulette); 4) suicides that occurred while under the influence of substances taken voluntarily; 5) suicides among decedents with mental health problems that affected their thinking, feelings, or mood (e.g., while experiencing an acute episode of a mental health condition, such as schizophrenia or other psychotic conditions, depression, or posttraumatic stress disorder); and 6) suicides involving another person who provided passive (only) assistance to the decedent (e.g., supplying the means or information needed to complete the act). This category does not include deaths caused by chronic or acute substance use without the intent to die, deaths attributed to autoerotic behavior (e.g., self- strangulation during sexual activity), or assisted suicides (legal or nonlegal). Corresponding ICD-10 codes included in NVDRS are X60–X84, Y87.0, and U03 (Box 1).
- **Homicide.** A homicide is a death resulting from the use of physical force or power, threatened or actual, against another person, group, or community when a preponderance of evidence indicates that the use of force was intentional. Two special scenarios that CDC’s National Center for Health Statistics regards as homicides are included in the NVDRS case definition: 1) arson with no specified intent to injure someone and 2) a stabbing with intent unspecified. This category also includes the following scenarios: 1) deaths when the suspect intended to only injure rather than kill the victim, 2) deaths resulting from a heart attack induced when the suspect used force or power against the victim, 3) deaths that occurred when a person killed an attacker in self-defense, 4) deaths resulting from a weapon that discharged unintentionally while being used to control or frighten the victim, 5) deaths attributed to child abuse without intent being specified, 6) deaths attributed to an intentional act of neglect by one person against another, 7) deaths of liveborn infants that resulted from a direct injury because of violence sustained before birth, and 8) deaths identified as a justifiable homicide when the person committing homicide was not a law enforcement officer. This category excludes vehicular homicide without intent to injure, unintentional poisoning deaths involving illegal or prescription drugs even when the person who provided drugs was charged with homicide, unintentional firearm injury deaths (a separate category in NVDRS), combat deaths or acts of war, deaths of unborn fetuses, and deaths of infants that resulted indirectly from violence sustained by the mother before birth (e.g., death from prematurity after premature labor brought on by violence). Corresponding ICD-10 codes included in NVDRS are X85–X99, Y00–Y09, Y87.1, and U01–U02 (Box 1).
- **Legal intervention.** A death from legal intervention is a death in which a person is killed or died as a result of injuries inflicted by a law enforcement officer or another peace officer (i.e., a person with specified legal authority to use deadly force), including military police, while acting in the line of duty. The term “legal intervention” is a classification from ICD-10 (Y35.0) and does not denote the lawfulness or legality of the circumstances surrounding a death caused by law enforcement. Legal intervention deaths also include a small subset of cases in which force was applied without clear lethal intent (e.g., during restraint or when applying force with a typically nondeadly weapon, such as a Taser) or in which the death occurred while the person was fleeing capture. This category excludes legal executions. Corresponding ICD-10 codes included in NVDRS are Y35.0–Y35.4, Y35.6, Y35.7, and Y89.0 (Box 1).
- **Unintentional firearm.** An unintentional firearm injury death is a death resulting from a penetrating injury or gunshot wound from a weapon that uses a powder charge to fire a projectile and for which a preponderance of evidence indicates that the shooting was not directed intentionally at the decedent with an intent to injure. Examples include the following: 1) a person who received a self-inflicted wound while playing with a firearm; 2) a person who mistakenly believed a firearm was unloaded and shot another person; 3) a child aged <6 years who shot himself or herself or another person; 4) a person who died as a result of a celebratory firing that was not intended to frighten, control, or harm anyone; 5) a person who unintentionally shot himself or herself when using a firearm to frighten, control, or harm another person; 6) a soldier who was shot during a field exercise but not in a combat situation; and 7) an infant who died after birth from an unintentional firearm injury that was sustained in utero. This category excludes injuries caused by unintentionally striking a person with the firearm (e.g., hitting a person on the head with the firearm rather than firing a projectile) and unintentional injuries from nonpowder guns (e.g., BB, pellet, or other compressed-air–powered or compressed-gas–powered guns). Corresponding ICD-10 codes included in NVDRS are W32–W34 and Y86 (Box 1).
- **Undetermined intent.** A death of undetermined intent is a death resulting from the use of force or power against oneself or another person for which the evidence indicating one manner of death is no more compelling than evidence indicating another. This category includes coroner or medical examiner rulings in which records from data providers indicate that investigators did not find enough evidence to determine whether the injury was intentional (e.g., unclear whether a drug overdose was unintentional or a suicide). Corresponding ICD-10 codes included in NVDRS are Y10–Y34, Y87.2, and Y89.9 (Box 1).

### Variables Analyzed

NVDRS collects approximately 600 unique variables for each death (Boxes 1, 2, and 3). The number of variables recorded for each incident depends on the content and completeness of the source documents. Variables in NVDRS include

BOX 2 **Methods used to inflict injury — National Violent Death Reporting System, 2021**

- Firearm: method that uses a powder charge to fire a projectile from the weapon (excludes BB gun, pellet gun, or compressed air- or gas-powered gun)
- Hanging, strangulation, or suffocation (e.g., hanging by the neck, manual strangulation, or plastic bag over the head)
- Poisoning (e.g., fatal ingestion or injection of an illegal drug, alcohol, pharmaceutical, carbon monoxide, gas, rat poison, or insecticide)
- Sharp instrument (e.g., knife, razor, machete, or pointed instrument)
- Blunt instrument (e.g., club, bat, rock, or brick)
- Fall: being pushed or jumping
- Motor vehicle (e.g., car, bus, motorcycle, or other transport vehicle)
- Other transport vehicle (e.g., train, plane, or boat)
- Personal weapons (e.g., hands, fists, or feet)
- Drowning: inhalation of liquid (e.g., in bathtub, lake, or other source of water or liquid)
- Fire or burns: inhalation of smoke or the direct effects of fire or chemical burns
- Shaking (e.g., shaken baby syndrome)
- Intentional neglect: starvation, lack of adequate supervision, or withholding of health care
- Explosive (e.g., bomb, rocket, or grenade)
- Nonpowder gun (e.g., BB, pellet, or compressed air- or gas-powered gun)
- Biologic weapons (e.g., anthrax, plague, or botulism)
- Other (single method): any method other than those already listed (e.g., electrocution or exposure to environment or weather)
- Unknown: method not reported or not known

BOX 3 Circumstances preceding* fatal injury, by manner of death — National Violent Death Reporting System, 2021

**All Manners of Death**

*Mental Health and Substance Use*

- Alcohol problem: decedent was perceived by self or others to have a problem with, or to be addicted to or dependent on, alcohol.
- Current depressed mood: decedent was perceived by self or others to be feeling depressed at the time of incident.
- Current diagnosed mental health problem: decedent was identified as having a mental health disorder or syndrome listed in the Diagnostic and Statistical Manual, Version 5 (DSM-5), with the exception of alcohol and other substance dependence (these are captured in separate variables).
- Current mental health or substance use treatment: decedent was receiving mental health or substance use treatment as evidenced by a current prescription for a psychotropic medication, visit or visits to a mental health professional, or participation in a therapy group or outpatient program within the previous 2 months.
- History of ever being treated for mental health or substance use problem: decedent was identified as having ever received mental health or substance use treatment.
- Nonadherence to treatment for a mental health or substance use problem: decedent did not actively participate in a prescribed regimen for their mental health or substance use treatment or did not follow a set treatment plan as recommended by a mental health or medical professional.
- Other substance use problem (excludes alcohol): decedent was perceived by self or others to have a problem with, or be addicted to or dependent on, a substance other than alcohol.
- Other addiction: decedent was perceived by self or others to have an addiction to or dependency on something other than to alcohol or other substance (e.g., gambling or sex).
- Type of mental health diagnosis: type of DSM-5 diagnosis reported for the decedent.

*Crime and Criminal Activity*

- Precipitated by another crime: incident occurred as the result of another serious crime.
- Crime in progress: another serious crime was in progress at the time of the incident.
- Nature of crime: the specific type of other crime that occurred precipitated the incident (e.g., sexual assault, gambling, robbery, or drug trafficking).

*Relationship and Life Stressors*

- Argument or conflict: a specific argument or disagreement led to the victim’s death.
- Caretaker abuse or neglect led to death: decedent was experiencing physical, sexual, or psychological abuse; physical (including medical or dental), emotional, or educational neglect; exposure to a violent environment; or inadequate supervision by a caretaker that led to death.
- Exposure to disaster: decedent was exposed to a disaster (e.g., earthquake, bombing, or COVID-19 pandemic) that was perceived as contributing to the incident.
- Family relationship problem: decedent was experiencing problems with a family member other than an intimate partner.
- Family stressor: decedent was experiencing problems related to the family home environment that are not related to relationship problems and involve family members other than intimate partners.
- History of child abuse or neglect: as a child, decedent had history of physical, sexual, or psychological abuse; physical (including medical or dental), emotional, or educational neglect; exposure to a violent environment; or inadequate supervision by a caretaker.
- Household known to local authorities: someone in the household, other than the decedent, had previous contact with local authorities.
- Living transition or loss of independent living: decedent recently transitioned from an independent or family living situation (e.g., family home or living on one’s own) to an assisted one, or that such a transition was imminent.
- Other relationship problem (nonintimate): decedent was experiencing problems with a friend or associate (other than an intimate partner or family member).
- Perpetrator of interpersonal violence during previous month: decedent perpetrated interpersonal violence during the past month.
- Physical fight (two persons, not a brawl): a physical fight between two persons that resulted in the death of the decedent, who was either involved in the fight, a bystander, or trying to stop the fight.
- Victim known to authorities: decedent had a history of contact with or was otherwise known to local, state, Federal, or international authorities.
- Victim of interpersonal violence during previous month: decedent was the target of interpersonal violence during the past month.

*Child Victim Incident*

- Previous Child Protective Services report on the child victim’s household: a Child Protective Services report was filed on the child decedent’s household before the fatal incident.
- Substance use problems in child victim’s household: evidence of substance use or misuse in child decedent’s household.

*Crisis Circumstances*

- Crisis during previous or upcoming 2 weeks: current crisis or acute precipitating event or events that either occurred during the previous 2 weeks or was impending in the following 2 weeks (e.g., a trial for a criminal offense begins the following week and appeared to have contributed to the death). Crises are associated with specific circumstance variables (e.g., alcohol problem was a crisis, or a family relationship problem was a crisis).
- Other crisis: a crisis related to a death but not captured by any of the standard circumstances.
**Suicide or Death of Undetermined Intent**
- Caregiver burden: stress or burden perceived by the decedent as a caregiver of a chronically ill, disabled, or elderly person appears to have contributed to the death.
- Disclosed suicidal intent: decedent had recently expressed suicidal feelings to another person with time for that person to intervene.
- Disclosed intent to whom: type of person (e.g., family member or current or former intimate partner) to whom the decedent recently disclosed suicidal thoughts or plans.
- Eviction or loss of housing: decedent was experiencing a recent or impending eviction or other loss of housing, or the threat of eviction or loss of housing.
- Financial problem: decedent was experiencing financial problems (e.g., bankruptcy, overwhelming debt, or foreclosure of a home or business).
- History of attempting suicide: decedent had previously attempted suicide before the fatal incident.
- History of nonsuicidal self-injury or self-harm: decedent had a history of intentionally inflicting pain or injuring one’s own body without the conscious intent of dying by suicide.
- History of suicidal thoughts or plans: decedent had previously expressed suicidal thoughts or plans.
- History of traumatic brain injury: decedent had history of traumatic brain injury.
- Intimate partner problem: decedent was experiencing problems with a current or former intimate partner.
- Job problem: decedent was either experiencing a problem at work or was having a problem with joblessness.
- Left a suicide note: decedent left a note, email message, video, or other communication indicating intent to die by suicide.
- Noncriminal legal problem: decedent was facing civil legal problems (e.g., a child custody or civil lawsuit).
- Physical health problem: decedent was experiencing physical health problems (e.g., a recent cancer diagnosis or chronic pain).
- Recent criminal legal problem: decedent was facing criminal legal problems (e.g., recent or impending arrest or upcoming criminal court date).
- School problem: decedent was experiencing a problem related to school (e.g., poor grades, bullying, social exclusion at school, or performance pressures).
- Suicide of family member or friend: decedent was distraught over, or reacting to, the suicide of a family member or friend.
- Other death of family member or friend: decedent was distraught over, or reacting to, the nonsuicide death of a family member or friend.
- Traumatic anniversary: the incident occurred on or near the anniversary of a traumatic event in the decedent’s life.

**Homicide or Legal Intervention Death**

- Brawl: mutual physical fight involving three or more persons.
- Drive-by shooting: suspect drove near the decedent and fired a weapon while driving or steps out of the car just long enough to use a weapon.
- Drug involvement: drug dealing, drug trade, or illegal drug use suspected to have played a role in precipitating the incident.
- Gang related: motive of the incident was gang related, or a gang member was a suspect or decedent in the incident.
- Hate crime: decedent was selected intentionally because of his or her actual or perceived gender, religion, sexual orientation, race, ethnicity, disability, immigrant status, or national origin.
- Intimate partner violence–related: incident is related to conflict between current or former intimate partners; includes all deaths of an intimate partner as well as others (e.g., child, parent, friend, or law enforcement officer) killed in an incident that originated in a conflict between intimate partners.
- Jealousy (lovers’ triangle): jealousy or distress over an intimate partner’s relationship or suspected relationship with another person.
- Justifiable self-defense: decedent was killed by a law enforcement officer in the line of duty or by a civilian in legitimate self-defense or in defense of others.
- Mentally ill suspect: suspect’s attack on decedent was believed to be the direct result of a mental health problem (e.g., schizophrenia or other psychotic condition, depression, or posttraumatic stress disorder).
- Mercy killing: decedent wished to die because of a terminal or hopeless disease or condition, and documentation indicates that the decedent wanted to be killed.
- Prostitution: prostitution or related activity that includes prostitutes, pimps, clients, or others involved in such activity.
- Random violence: decedent was killed in a random act of violence (i.e., an act in which the suspect is not concerned with who is being harmed, just that someone is being harmed).
- Stalking: pattern of unwanted harassing or threatening tactics by either the decedent or suspect.
- Victim used a weapon: decedent used a weapon to attack or defend during the course of the incident.
- Victim was a bystander: decedent was not the intended target in the incident (e.g., pedestrian walking past a gang fight).
- Victim was an intervener assisting a crime victim: decedent was attempting to assist a crime victim at the time of the incident (e.g., a child attempts to intervene and is killed while trying to assist a parent who is being assaulted).
- Victim was a police officer on duty: decedent was a law enforcement officer killed in the line of duty.
- Walk-by assault: decedent was killed by a targeted attack (e.g., ambush) where the suspect fled on foot.
*Child Victim Homicide Incident*
- Caregiver use of corporal punishment contributed to the death of the child victim: corporal punishment (i.e., physical punishment with or without an implement that is intended to punish or discipline a child) by the child’s caregiver contributed to the death of the child decedent.

**Unintentional Firearm Injury Death**

*Context of Injury*

- Celebratory firing: shooter fired firearm in celebratory manner (e.g., firing into the air at midnight on New Year’s Eve).
- Cleaning firearm: shooter pulled trigger or firearm discharged while cleaning, repairing, assembling, or disassembling firearm.
- Hunting: death occurred any time after leaving home for a hunting trip and before returning home from a hunting trip.
- Loading or unloading firearm: firearm discharged when the shooter was loading or unloading ammunition.
- Playing with firearm: shooter was playing with a firearm when it discharged.
- Showing firearm to others: firearm was being shown to another person when it discharged, or the trigger was pulled.
- Target shooting: shooter was aiming for a target and unintentionally hit the decedent; can be at a shooting range or an informal backyard setting (e.g., teenagers shooting at signposts on a fence).
- Other context of injury: shooting occurred during some context other than those already described.
*Mechanism of Injury*
- Bullet ricocheted: bullet ricocheted from its intended target and struck the decedent.
- Firearm fired due to defect or malfunction: firearm had a defect or malfunctioned as determined by a trained firearm examiner.
- Firearm fired while holstering: firearm was being replaced or removed from holster or clothing.
- Firearm fired while operating safety or lock: shooter unintentionally fired the firearm while operating the safety or lock.
- Firearm was dropped: firearm discharged when it was dropped.
- Firearm was mistaken for toy: firearm was mistaken for a toy and was fired without the user understanding the danger.
- Thought firearm safety was engaged: shooter thought the safety was on and firearm would not discharge.
- Thought unloaded, magazine disengaged: shooter thought the firearm was unloaded because the magazine was disengaged.
- Thought firearm was unloaded, other reason: shooter thought the firearm was unloaded for reason other than magazine disengaged or for an unspecified reason.
- Unintentionally pulled trigger: shooter unintentionally pulled the trigger (e.g., while grabbing the firearm or holding it too tightly).
- Other mechanism of injury: shooting occurred as the result of a mechanism not already described.

* Circumstances preceding death are defined as the events that precipitated, occurred during, or otherwise contributed to the infliction of a fatal injury as identified by investigators.

- manner of death (i.e., the intent to cause death [suicide, homicide, legal intervention, unintentional, and undetermined] of the person on whom a fatal injury was inflicted) (Box 1);
- demographic information (e.g., age, sex, and race and ethnicity) of victims and suspects (if applicable);
- method of injury (i.e., the mechanism used to inflict a fatal injury) (Box 2);
- location, date, and time of injury and death;
- toxicology findings (for decedents who were tested);
- circumstances (i.e., the events that preceded, precipitated, or occurred during or otherwise contributed to the fatal incident as identified by investigators as relevant and therefore might have contributed to the infliction of a fatal injury) (Box 3);
- whether the decedent was a victim (i.e., a person who died as a result of a violence-related injury) or both a suspect and a victim (i.e., a person believed to have inflicted a fatal injury on a victim who then was fatally injured, such as the perpetrator of a homicide followed by suicide);
- information about any known suspects (i.e., a person or persons believed to have inflicted a fatal injury on a victim);
- incident (i.e., an occurrence in which one or more persons sustained a fatal injury that was linked to a common event or perpetrated by the same suspect or suspects during a 24-hour period); and
- type of incident (i.e., a combination of the manner of death and whether single or multiple victims were involved in an incident).

### Circumstances Preceding Death

Circumstances preceding death are defined as the events that precipitated, occurred during, or otherwise contributed to the infliction of a fatal injury as identified by investigators (Box 3). Circumstances are reported based on the content of coroner or medical examiner and law enforcement investigative reports. Certain circumstances are coded to a specific manner of death (e.g., “history of suicidal thoughts or plans” is collected for suicides and deaths of undetermined intent); other circumstances are coded across all manners of death (e.g., “ever treated for mental health or substance use problem”). The data abstractor selects from a list of potential circumstances and is required to code all circumstances that are known to relate to each incident. If circumstances are unknown (e.g., a body found in the woods with no other details reported), the data abstractor does not endorse circumstances; these deaths are then excluded from the denominator for circumstance values. If either the coroner or medical examiner report or law enforcement report indicates the presence of a circumstance, then the abstractor endorses the circumstance. For example, if a law enforcement report indicates that a decedent had disclosed thoughts of suicide or an intent to die by suicide, then the circumstance variable “recent disclosure of suicidal thoughts or intent” is endorsed.

Data abstractors draft two incident narratives: one that summarizes the sequence of events of the incident from the perspective of the coroner or medical examiner record and one that summarizes the sequence of events of the incident from the perspective of the law enforcement report. In addition to briefly summarizing the incident (i.e., the who, what, when, where, and why of the incident), the narratives provide supporting information, context, and details on circumstances indicated by the data abstractor for understanding the incident, record information and additional detail that cannot be captured elsewhere, and facilitate data quality control checks on the coding of key variables. This report introduces additional incident and circumstance variables from previous years, including child victim–specific circumstance information (e.g., previous Child Protective Services report on victim’s household, substance use in decedent’s home, and corporal punishment).

### Coding Training and Quality Control

Ongoing coding support for data abstractors is provided by CDC through an electronic help desk, monthly conference calls, annual in-person or virtual meetings that include coding training for data abstractors, and regular technical assistance conference calls with individual VDRS programs. In addition, all data abstractors are invited to participate in monthly coding work group calls. VDRS programs can conduct additional abstractor training workshops and activities at their own discretion, including through the use of NVDRS Data Abstractor eLearn Training Modules. An NVDRS coding manual (*8*) with CDC-issued standard guidance on coding criteria and examples for each data element is provided to each VDRS program and is publicly available (https://www.cdc.gov/nvdrs/resources/nvdrscodingmanual.pdf). Software features that enhance coding reliability include automated validation rules and a hover-over feature containing variable-specific information.

Each year, VDRS programs are required to reabstract at least 5% of cases using multiple abstractors to identify inconsistencies. In addition, each VDRS program’s data quality plan is evaluated by CDC. Before the data are released each year, CDC conducts a quality control analysis that involves the review of multiple variables for data inconsistencies, with special focus on abstractor-assigned variables (e.g., method of injury and manner of death). If CDC finds inconsistencies, the VDRS program is notified and asked for a response or correction. VDRS programs must meet CDC standards for completeness of circumstance data to be included in the national data set. VDRS programs must have circumstance information abstracted from either the coroner or medical examiner record or the law enforcement report for at least 50% of deaths. However, VDRS programs often exceed this requirement. For 2021, a total of 79.6% of suicides, homicides, and legal intervention deaths in NVDRS had circumstance data from either the coroner or medical examiner record or the law enforcement report. In addition, core variables that represent demographic characteristics (e.g., age, sex, and race and ethnicity) and manners of death were missing or unknown for <0.1% of cases. To ensure the final data set has no duplicate records, during the data closeout process, NVDRS first identifies any records within VDRS programs that match on a subset of 14 key variables and then asks VDRS programs to review these records to determine whether they are true duplicates. One record in any set of two or more records that are true duplicates is retained, and the others are deleted by the VDRS program. Next, NVDRS uses SAS software (version 9.4; SAS Institute) to search for any instances of duplicates of a unique identification variable associated with each decedent record. As a third and final check for duplicates, the SAS data set is created with an index that only executes successfully if no duplicates of this identification variable are found.

### Time Frame

VDRS programs are required to begin entering each death into the web-based system within 4 months from the date the death occurred. VDRS programs have an additional 16 months from the end of the calendar year in which the death occurred to complete each incident record. Although VDRS programs typically meet timeliness requirements, additional details about an incident occasionally arrive after a deadline has passed. New incidents also might be identified after the deadline (e.g., when a death certificate is revised, new evidence is obtained that changes a manner of death, or an ICD-10 misclassification is corrected to meet the NVDRS case definition). These additional data are incorporated on an ongoing basis into NVDRS when analysis files are updated in real time in the web-based system; 5 months after the 16-month data collection period for the 2021 data year, case counts increased by <0.1%.

### Inclusion Criteria

The inclusion criteria for deaths in this report are as follows: 1) cases met the NVDRS case definition; 2) cases occurred in states and jurisdictions participating in NVDRS in 2021; and 3) at least 50% of cases for each included state, district, territory, or subset of counties had circumstance information collected from the coroner or medical examiner record or law enforcement report. Data for Florida and Hawaii were ineligible to be included in this report because data did not meet the completeness threshold for circumstances.

Of the participating VDRS programs, 46 states (Alabama, Alaska, Arizona, Arkansas, Colorado, Connecticut, Delaware, Georgia, Idaho, Illinois, Indiana, Iowa, Kansas, Kentucky, Louisiana, Maine, Maryland, Massachusetts, Michigan, Minnesota, Mississippi, Missouri, Montana, Nebraska, Nevada, New Hampshire, New Jersey, New Mexico, New York, North Carolina, North Dakota, Ohio, Oklahoma, Oregon, Pennsylvania, Rhode Island, South Carolina, South Dakota, Tennessee, Utah, Vermont, Virginia, Washington, West Virginia, Wisconsin, and Wyoming) collected information on all violent deaths that occurred in their state in 2021. In addition, data were collected on deaths that occurred in the District of Columbia and Puerto Rico in 2021. Two states, California and Texas, joined NVDRS with plans to collect data on deaths in a subset of counties. California collected data from death certificates for violent deaths in the state in 2021 (n = 7,020); data for violent deaths that occurred in 31 counties (Amador, Butte, Colusa, Fresno, Glenn, Humboldt, Imperial, Kings, Lake, Lassen, Los Angeles, Mendocino, Merced, Modoc, Mono, Orange, Placer, Sacramento, San Benito, San Diego, San Francisco, San Luis Obispo, San Mateo, Santa Cruz, Shasta, Siskiyou, Solano, Sonoma, Tehama, Ventura, and Yolo) also included information from coroner or medical examiner records and law enforcement reports and are included throughout the rest of the report (n = 4,319; 61.5%). These 31 counties represented 64% of California’s population (*9*). Texas also collected data from death certificates for all violent deaths in the state in 2021 (n = 6,990); data for deaths that occurred in 13 counties (Bell, Bexar, Collin, Dallas, Denton, El Paso, Fort Bend, Harris, Montgomery, Nueces, Tarrant, Travis, and Williamson) also included information from coroner or medical examiner records and law enforcement reports and are included throughout the rest of the report (n = 4,326; 61.9%). These 13 counties represented 63% of the state’s population (*9*). Because <100% of deaths were abstracted, data from California and Texas do not represent all cases of matching deaths occurring in these states.

### Analyses

This report includes data for deaths that occurred in 48 states (46 states collecting statewide data, 31 California counties, and 13 Texas counties), the District of Columbia, and Puerto Rico in 2021. VDRS program-level data received by CDC as of May 24, 2023, were consolidated and analyzed. The numbers, percentages, and crude rates are presented in aggregate for all deaths by the abstractor-assigned manner of death. The suicide rate was calculated using denominators among populations aged ≥10 years. The rates for other manners of death used denominators among populations of all ages. The rates for cells with frequency <20 are not reported because of the instability of those rates. Denominators for the rates for the two states that did not collect statewide data (California and Texas) correspond to the populations of the counties from which data were collected. The rates could not be calculated for certain variables (e.g., circumstances) because denominators were unknown.

The U.S. Census Bureau made recent improvements to their collection of race and ethnicity data beginning with Census 2020 data collection. The U.S. Census Bureau transitioned from bridged-race population estimates to single race estimates with six categories: AI/AN, Asian, Black, NH/PI, White, and more than one race. These county-level population estimates for 2021 were used as denominators in the crude rate calculations for the 48 states (46 states collecting statewide data, 31 California counties, and 13 Texas counties), and the District of Columbia (*10*). Data for Puerto Rico were analyzed separately, as the rates specific to race and ethnicity are not available for Puerto Rico because the U.S. Census Bureau estimates for Puerto Rico do not include race or Hispanic or Latino (Hispanic) origin (*11*). Population estimates by sex and age were used as denominators in the crude rate calculations for Puerto Rico (*12*).

## Results

### Violent Deaths in 48 States and the District of Columbia

For 2021, a total of 48 NVDRS states (46 states collecting statewide data, 31 California counties, and 13 Texas counties) and the District of Columbia collected data on 68,866 incidents involving 70,688 deaths, which accounted for 86.5% of all homicides, legal intervention deaths, suicides, unintentional firearm injury deaths, and deaths of undetermined intent in the United States during this period. (Supplementary Table 1, https://stacks.cdc.gov/view/cdc/157365). Suicides (n = 41,116; 58.2%) accounted for the highest rate of deaths captured by NVDRS (16.4 per 100,000 population aged ≥10 years). The homicide rate was 7.9 per 100,000 population (n = 22,288; 31.5%). Deaths of undetermined intent (n = 5,806; 8.2%), legal intervention deaths (n = 923; 1.3%), and unintentional firearm injury deaths (n = 555; <1.0%) occurred at lower rates (2.0, 0.3, and 0.2 per 100,000 population, respectively). Data for deaths by manner that include statewide counts and the rates for California and Texas are available (Supplementary Table 2, https://stacks.cdc.gov/view/cdc/157365). More than half of NVDRS deaths (59.2%) involved firearms as the method of injury, and the majority of victims (60.4%) were injured in a house or apartment (Supplementary Table 3, https://stacks.cdc.gov/view/cdc/157365).

### Suicides

#### Sex, Age Group, and Race and Ethnicity

For 2021, a total of 48 NVDRS states (46 states collecting statewide data, 31 California counties, and 13 Texas counties) and the District of Columbia collected data on 41,082 incidents involving 41,116 suicide deaths among persons aged ≥10 years (Supplementary Table 1, https://stacks.cdc.gov/view/cdc/157365). The overall suicide rate was 16.4 per 100,000 population aged ≥10 years (Table 1).

**TABLE 1:** Number, percentage,* and rate^†^ of suicides among persons aged ≥10 years,^§^ by selected demographic characteristics of decedent,^¶^ method used, location in which injury occurred, and incident characteristics — National Violent Death Reporting System, 48 states and District of Columbia,** 2021

Characteristic	Male		Female		Total	
	No. (%)	Rate	No. (%)	Rate	No. (%)	Rate
**Age group, yrs**						
10–14	310 (<1.0)	3.3	208 (2.5)	2.3	**518 (1.3)**	**2.8**
15–19	1,560 (4.8)	16.5	479 (5.8)	5.3	**2,040 (5.0)**	**11.1**
20–24	3,029 (9.2)	32.2	620 (7.5)	6.8	**3,651 (8.9)**	**19.8**
25–29	3,146 (9.6)	32.2	696 (8.4)	7.3	**3,844 (9.3)**	**20.0**
30–34	3,096 (9.4)	31.0	708 (8.5)	7.2	**3,804 (9.3)**	**19.2**
35–44	5,388 (16.4)	28.9	1,392 (16.8)	7.5	**6,780 (16.5)**	**18.3**
45–54	4,854 (14.8)	28.0	1,517 (18.3)	8.7	**6,371 (15.5)**	**18.3**
55–64	4,722 (14.4)	26.3	1,408 (17.0)	7.5	**6,130 (14.9)**	**16.7**
65–74	3,482 (10.6)	25.8	797 (9.6)	5.3	**4,279 (10.4)**	**14.9**
75–84	2,238 (6.8)	37.5	351 (4.2)	4.6	**2,589 (6.3)**	**19.1**
≥85	998 (3.0)	55.7	111 (1.3)	3.5	**1,109 (2.7)**	**22.2**
Unknown	1 (<1.0)	—^††^	0 (—)	—	**1 (<1.0)**	—
**Race and ethnicity^§§^**						
American Indian or Alaska Native	448 (1.4)	45.8	154 (1.9)	15.2	**602 (1.5)**	**30.2**
Asian	772 (2.4)	11.3	324 (3.9)	4.3	**1,096 (2.7)**	**7.7**
Black or African American	2,655 (8.1)	17.5	630 (7.6)	3.8	**3,285 (8.0)**	**10.3**
Native Hawaiian or other Pacific Islander	54 (<1.0)	30.7	12 (<1.0)	—	**66 (<1.0)**	**18.9**
White	25,411 (77.4)	32.6	6,299 (76.0)	7.9	**31,714 (77.1)**	**20.1**
More than one race	379 (1.2)	15.9	120 (1.4)	4.8	**499 (1.2)**	**10.3**
Hispanic or Latino	2,937 (8.9)	15.0	697 (8.4)	3.7	**3,635 (8.8)**	**9.4**
Unspecified or unknown	168 (<1.0)	—	51 (<1.0)	—	**219 (<1.0)**	—
**Method**						
Firearm	19,526 (59.5)	15.8	2,795 (33.7)	2.2	**22,322 (54.3)**	**8.9**
Hanging, strangulation, or suffocation	8,334 (25.4)	6.8	2,430 (29.3)	1.9	**10,765 (26.2)**	**4.3**
Poisoning	2,179 (6.6)	1.8	2,216 (26.7)	1.8	**4,396 (10.7)**	**1.8**
Fall	802 (2.4)	0.7	272 (3.3)	0.2	**1,074 (2.6)**	**0.4**
Sharp instrument	707 (2.2)	0.6	150 (1.8)	0.1	**857 (2.1)**	**0.3**
Motor vehicles (e.g., buses, motorcycles, or other transport vehicles)	508 (1.5)	0.4	168 (2.0)	0.1	**677 (1.6)**	**0.3**
Drowning	245 (<1.0)	0.2	108 (1.3)	<0.1	**354 (<1.0)**	**0.1**
Fire or burns	123 (<1.0)	0.1	36 (<1.0)	<0.1	**159 (<1.0)**	**<0.1**
Blunt instrument	78 (<1.0)	<0.1	18 (<1.0)	—	**96 (<1.0)**	**<0.1**
Other (e.g., Taser, electrocution, nail gun, intentional neglect, or personal weapon)	46 (<1.0)	—	13 (<1.0)	—	**59 (<1.0)**	—
Unknown	276 (<1.0)	—	81 (<1.0)	—	**357 (<1.0)**	—
**Location of injury**						
House or apartment	23,011 (70.1)	18.7	6,312 (76.2)	5.0	**29,325 (71.3)**	**11.7**
Motor vehicle	1,884 (5.7)	1.5	392 (4.7)	0.3	**2,276 (5.5)**	**0.9**
Natural area	1,546 (4.7)	1.3	269 (3.2)	0.2	**1,816 (4.4)**	**0.7**
Street or highway	987 (3.0)	0.8	176 (2.1)	0.1	**1,163 (2.8)**	**0.5**
Hotel or motel	700 (2.1)	0.6	246 (3.0)	0.2	**946 (2.3)**	**0.4**
Parking lot, public garage, or public transport	606 (1.8)	0.5	111 (1.3)	<0.1	**718 (1.7)**	**0.3**
Jail or prison	610 (1.9)	0.5	55 (<1.0)	<0.1	**665 (1.6)**	**0.3**
Park, playground, or sports or athletic area	587 (1.8)	0.5	70 (<1.0)	<0.1	**657 (1.6)**	**0.3**
Bridge	256 (<1.0)	0.2	73 (<1.0)	<0.1	**330 (<1.0)**	**0.1**
Commercial or retail area	281 (<1.0)	0.2	35 (<1.0)	<0.1	**316 (<1.0)**	**0.1**
Railroad tracks	194 (<1.0)	0.2	62 (<1.0)	<0.1	**256 (<1.0)**	**0.1**
Supervised residential facility	167 (<1.0)	0.1	58 (<1.0)	<0.1	**225 (<1.0)**	**<0.1**
Other location^¶¶^	1,136 (3.5)	—	185 (2.2)	—	**1,321 (3.2)**	—
Unknown	859 (2.6)	—	243 (2.9)	—	**1,102 (2.7)**	—
**Incident**						
Emergency medical services present	22,119 (67.4)	18.0	5,820 (70.2)	4.6	**27,942 (68.0)**	**11.2**
Injured at victim’s home	20,864 (63.6)	16.9	5,748 (69.4)	4.5	**26,614 (64.7)**	**10.6**
Victim was suspected of alcohol use preceding the incident	5,145 (15.7)	4.2	1,208 (14.6)	1.0	**6,353 (15.5)**	**2.5**
Victim was recently released from or admitted to an institutional setting	1,995 (6.1)	1.6	540 (6.5)	0.4	**2,535 (6.2)**	**1.0**
Child present or witnessed incident	1,564 (4.8)	1.3	476 (5.7)	0.4	**2,041 (5.0)**	**0.8**
Victim was in public custody when injury occurred	1,033 (3.1)	0.8	106 (1.3)	<0.1	**1,139 (2.8)**	**0.5**
Victim was experiencing housing instability	900 (2.7)	0.7	204 (2.5)	0.2	**1,104 (2.7)**	**0.4**
Victim was experiencing homelessness	486 (1.5)	0.4	103 (1.2)	<0.1	**589 (1.4)**	**0.2**
Victim was injured at work or while working	303 (<1.0)	0.3	28 (<1.0)	<0.1	**331 (<1.0)**	**0.1**
**Total**	**32,824 (100)**	**26.6**	**8,287 (100)**	**6.5**	**41,116 (100)**	**16.4**

* Percentages might not total 100% due to rounding.

^†^ Per 100,000 population aged ≥10 years.

^§^ Suicide is not reported for decedents aged <10 years because of the small number of suicide deaths per year in this age group.

^¶^ Sex was unknown for five decedents.

** Includes all U.S. states and District of Columbia, with exception of Florida and Hawaii. Data for California are for violent deaths that occurred in 31 counties (Amador, Butte, Colusa, Fresno, Glenn, Humboldt, Imperial, Kings, Lake, Lassen, Los Angeles, Mendocino, Merced, Modoc, Mono, Orange, Placer, Sacramento, San Benito, San Diego, San Francisco, San Luis Obispo, San Mateo, Santa Cruz, Shasta, Siskiyou, Solano, Sonoma, Tehama, Ventura, and Yolo). Data for Texas are for violent deaths that occurred in 13 counties (Bell, Bexar, Collin, Denton, El Paso, Fort Bend, Dallas, Harris, Montgomery, Nueces, Tarrant, Travis, and Williamson). Denominators for the rates for California and Texas represent only the populations of the counties from which the data were collected.

^††^ Rate is not reported when number of decedents is <20 or when characteristic response is “Other” or “Unknown.”

^§§^ Persons of Hispanic or Latino (Hispanic) origin might be of any race but are categorized as Hispanic; all racial groups are non-Hispanic.

^¶¶^ Other location includes (in descending order): hospital or medical facility; cemetery, graveyard, or other burial ground; industrial or construction area; preschool, school, college, or school bus; farm; office building; abandoned house, building, or warehouse; synagogue, church, or temple; bar or nightclub; and other unspecified location.

The overall suicide rate for males (26.6 per 100,000 population aged ≥10 years) was 4.1 times the rate for females (6.5 per 100,000 population aged ≥10 years) (Table 1). The suicide rate for males ranged from 1.4 to 15.9 times the rate for females across age groups and 2.6 to 4.6 times the rate for females across racial and ethnic groups. Adults aged ≥85 years (22.2 per 100,000 population aged ≥10 years), 25–29 years (20.0 per 100,000 population aged ≥10 years), and 20–24 years (19.8 per 100,000 population aged ≥10 years) had the highest rates of suicide across age groups. White persons accounted for most (77.1%) of suicides; however, AI/AN persons had the highest rate of suicide (30.2 per 100,000 population aged ≥10 years) among all racial and ethnic groups.

Among male suicide decedents, nearly one half (45.6%) were aged 35–64 years (Table 1). By age group, men aged ≥85 years had the highest rate of suicide (55.7 per 100,000 population aged ≥10 years), followed by men aged 75–84 years (37.5 per 100,000 population aged ≥10 years) and 20–24 and 25–29 years (both 32.2 per 100,000 population aged ≥10 years). Across racial and ethnic groups, AI/AN males had the highest rate of suicide (45.8 per 100,000 population aged ≥10 years), followed by White males (32.6 per 100,000 population aged ≥10 years) and NH/PI males (30.7 per 100,000 population aged ≥10 years). The rate of suicide for AI/AN males was 4.1 times the rate for males with the lowest rate (non-Hispanic Asian [Asian]; 11.3 per 100,000 population aged ≥10 years). The suicide rate was 17.5 per 100,000 population aged ≥10 years for Black males, 15.9 per 100,000 population aged ≥10 years for males of more than one race, and 15.0 per 100,000 population aged ≥10 years for Hispanic males.

Among females, those aged 35–64 years accounted for 52.1% of suicides (Table 1). Females aged 45–54 years had the highest rate of suicide (8.7 per 100,000 population aged ≥10 years), followed by those aged 35–44 years and 55–64 years (both 7.5 per 100,000 population). The suicide rate was highest among AI/AN females (15.2 per 100,000 population aged ≥10 years), followed by White females (7.9 per 100,000 population aged ≥10 years), persons of more than one race (4.8 per 100,000 population aged ≥10 years), and Asian females (4.3 per 100,000 population aged ≥10 years). The suicide rate for AI/AN females was 4.1 times the rate for females with the lowest rate (Hispanic females).

#### Method and Location of Injury

A firearm was used in more than one half (54.3%; 8.9 per 100,000 population aged ≥10 years) of suicides, followed by hanging, strangulation, or suffocation (26.2%; 4.3 per 100,000 population aged ≥10 years) and poisoning (10.7%; 1.8 per 100,000 population aged ≥10 years) (Table 1). Among males, the most common method of injury was a firearm (59.5%), followed by hanging, strangulation, or suffocation (25.4%). Among females, firearm (33.7%) was also the most common method of injury, followed by hanging, strangulation, or suffocation (29.3%) and poisoning (26.7%). Among all suicide decedents, the most common location of suicide was a house or apartment (71.3%), followed by a motor vehicle (5.5%), a natural area (4.4%), a street or highway (2.8%), and a hotel or motel (2.3%).

#### Incident Characteristics

Emergency medical services were present for a large percentage of suicide decedents (68.0%) (Table 1). Suicide decedents were commonly injured at their own homes (64.7%). Male and female suicide decedents had similar percentages of suspected alcohol use at the time of their death (15.7% and 14.6%, respectively). A child was either present or witnessed the incident for 5.0% of suicide decedents. A small proportion of suicide decedents were experiencing housing instability (2.7%) or homelessness (1.4%) at the time of death or were recently released from an institutional setting (6.2%).

#### Toxicology Results of Decedent

Toxicology tests for blood alcohol concentration (BAC) were conducted for 45.5% of suicide decedents (Table 2). Among those with positive results for alcohol (40.2%), 65.1% had a BAC ≥0.08 g/dL. Tests for the following substances were conducted for the percentage of decedents indicated in parentheses: amphetamines (36.8%), antidepressants (24.0%), barbiturates (30.5%), benzodiazepines (36.5%), cannabis (commonly referred to as marijuana; 33.8%), cocaine (35.8%), and opioids (38.5%). Positive results were found for 18.1% of decedents tested for amphetamines. Among those tested for antidepressants, 35.7% had positive results at the time of death; 1.8% of those tested for barbiturates had positive results, 20.6% of those tested for benzodiazepines had positive results, 29.8% of those tested for cannabis had positive results, and 6.9% of those tested for cocaine had positive results. Test results for opioids (including illegal and prescription opioids) were positive for 22.2% of decedents tested for these substances. Carbon monoxide was tested for a substantially smaller proportion of decedents (3.1%) but was identified in approximately one third of those decedents (41.7%).

**TABLE 2:** Number* and percentage of suicide decedents tested for alcohol and drugs and whose results were positive,^†^ by toxicology variable — National Violent Death Reporting System, 48 states and District of Columbia,^§^ 2021

Toxicology variable	Tested	Positive
	No. (%)	No. (%)
Blood alcohol concentration^¶^	18,702 (45.5)	7,513 (40.2)
Blood alcohol <0.08 g/dL	—	2,163 (28.8)
Blood alcohol ≥0.08 g/dL	—	4,890 (65.1)
Blood alcohol positive, level unknown	—	460 (6.1)
Amphetamines	15,112 (36.8)	2,729 (18.1)
Anticonvulsants	7,907 (19.2)	1,497 (18.9)
Antidepressants	9,868 (24.0)	3,526 (35.7)
Antipsychotics	7,395 (18.0)	986 (13.3)
Barbiturates	12,531 (30.5)	225 (1.8)
Benzodiazepines	14,989 (36.5)	3,085 (20.6)
Carbon monoxide	1,260 (3.1)	525 (41.7)
Cannabis**	13,902 (33.8)	4,149 (29.8)
Cocaine	14,724 (35.8)	1,009 (6.9)
Muscle relaxants	7,592 (18.5)	423 (5.6)
Opioids	15,839 (38.5)	3,523 (22.2)
Other drugs or substances^††^	2,781 (6.8)	2,486 (89.4)

* Number of suicide decedents = 41,116.

^†^ Percentage is of decedents tested for toxicology. Denominator for the percentage positive is based on the percentage tested.

^§^ Includes all U.S. states and District of Columbia, with exception of Florida and Hawaii. Data for California are for violent deaths that occurred in 31 counties (Amador, Butte, Colusa, Fresno, Glenn, Humboldt, Imperial, Kings, Lake, Lassen, Los Angeles, Mendocino, Merced, Modoc, Mono, Orange, Placer, Sacramento, San Benito, San Diego, San Francisco, San Luis Obispo, San Mateo, Santa Cruz, Shasta, Siskiyou, Solano, Sonoma, Tehama, Ventura, and Yolo). Data for Texas are for violent deaths that occurred in 13 counties (Bell, Bexar, Collin, Dallas, Denton, El Paso, Fort Bend, Harris, Montgomery, Nueces, Tarrant, Travis, and Williamson).

^¶^ Blood alcohol concentration of ≥0.08 g/dL is over the legal limit in all states and the District of Columbia and is used as the standard for intoxication.

** More commonly referred to as marijuana.

^††^ Other drugs or substances indicated if any results were positive; levels for these drugs or substances are not measured.

#### Precipitating Circumstances

Circumstances from coroner or medical examiner records and law enforcement reports were identified in 34,692 (84.4%) suicides (Table 3). Among decedents who had known circumstances, a mental health problem was the most common circumstance identified, with approximately half (49.0%) of decedents having a current diagnosed mental health problem and 29.2% experiencing a depressed mood at the time of death. Among the 16,994 decedents with a current diagnosed mental health problem, depression or dysthymia (71.9%), anxiety disorder (23.4%), and bipolar disorder (14.0%) were the most common diagnoses. Alcohol use problems were reported for 17.9% of suicide decedents, and other substance use problems (unrelated to alcohol) were reported for 18.0% of suicide decedents. Among suicide decedents, 22.9% were receiving mental health or substance use treatment at the time of death and 31.4% had a history of having been treated for a mental health or substance use problem (Table 3).

**TABLE 3:** Number* and percentage^†^ of suicides among persons aged ≥10 years,^§^ by decedent’s sex and precipitating circumstances — National Violent Death Reporting System, 48 states and District of Columbia,^¶^ 2021

Precipitating circumstance	Male	Female	Total
	No. (%)	No. (%)	No. (%)
**Mental health and substance use**			
Current diagnosed mental health problem**	12,391 (44.9)	4,598 (64.9)	**16,994 (49.0)**
Depression or dysthymia	8,738 (70.5)	3,483 (75.8)	**12,225 (71.9)**
Anxiety disorder	2,620 (21.1)	1,358 (29.5)	**3,980 (23.4)**
Bipolar disorder	1,584 (12.8)	800 (17.4)	**2,384 (14.0)**
Schizophrenia	938 (7.6)	243 (5.3)	**1,181 (6.9)**
Posttraumatic stress disorder	835 (6.7)	220 (4.8)	**1,057 (6.2)**
Attention deficit disorder or attention deficit hyperactivity disorder	469 (3.8)	102 (2.2)	**571 (3.4)**
Dementia	239 (1.9)	59 (1.3)	**298 (1.8)**
Autism spectrum	98 (<1.0)	15 (<1.0)	**113 (<1.0)**
Obsessive compulsive disorder	76 (<1.0)	32 (<1.0)	**108 (<1.0)**
Eating disorder	10 (<1.0)	24 (<1.0)	**34 (<1.0)**
Fetal alcohol syndrome	1 (<1.0)	2 (<1.0)	**3 (<1.0)**
Down syndrome	1 (<1.0)	0 (—)	**1 (<1.0)**
Other	547 (4.4)	214 (4.7)	**761 (4.5)**
Unknown	923 (7.4)	343 (7.5)	**1,267 (7.5)**
History of ever being treated for a mental health or substance use problem	7,701 (27.9)	3,197 (45.1)	**10,901 (31.4)**
Current depressed mood	7,984 (28.9)	2,143 (30.2)	**10,128 (29.2)**
Current mental health or substance use treatment	5,396 (19.6)	2,531 (35.7)	**7,929 (22.9)**
Substance use problem (excludes alcohol)	4,914 (17.8)	1,338 (18.9)	**6,252 (18.0)**
Alcohol problem	5,121 (18.6)	1,072 (15.1)	**6,193 (17.9)**
Non-adherence to mental health or substance use treatment	1,227 (4.4)	393 (5.5)	**1,621 (4.7)**
Other addiction (e.g., gambling or sexual)	222 (<1.0)	43 (<1.0)	**265 (<1.0)**
**Interpersonal factor**			
Intimate partner problem	7,056 (25.6)	1,603 (22.6)	**8,659 (25.0)**
Family relationship problem	1,904 (6.9)	692 (9.8)	**2,597 (7.5)**
Other death (not suicide) of family member or friend	1,749 (6.3)	506 (7.1)	**2,255 (6.5)**
Other relationship problem (nonintimate and nonfamily)	597 (2.2)	176 (2.5)	**773 (2.2)**
Suicide of family member or friend	549 (2.0)	192 (2.7)	**741 (2.1)**
Perpetrator of interpersonal violence during past month	681 (2.5)	54 (<1.0)	**735 (2.1)**
Victim of interpersonal violence during past month	50 (<1.0)	72 (1.0)	**122 (<1.0)**
**Life stressor**			
Crisis during previous or upcoming 2 weeks	8,550 (31.0)	1,904 (26.9)	**10,454 (30.1)**
Physical health problem	5,574 (20.2)	1,328 (18.7)	**6,902 (19.9)**
Argument or conflict	4,297 (15.6)	1,139 (16.1)	**5,437 (15.7)**
Job problem	2,322 (8.4)	400 (5.6)	**2,723 (7.8)**
Recent criminal legal problem	2,175 (7.9)	188 (2.7)	**2,363 (6.8)**
Financial problem	1,513 (5.5)	344 (4.9)	**1,857 (5.4)**
Family stressor	892 (3.2)	294 (4.1)	**1,186 (3.4)**
Exposure to disaster	822 (3.0)	223 (3.1)	**1,046 (3.0)**
Household had contact with local authorities	682 (2.5)	247 (3.5)	**929 (2.7)**
Noncriminal legal problem	676 (2.4)	180 (2.5)	**856 (2.5)**
Eviction or loss of home	673 (2.4)	170 (2.4)	**844 (2.4)**
School problem	350 (1.3)	125 (1.8)	**475 (1.4)**
History of traumatic brain injury	353 (1.3)	64 (<1.0)	**418 (1.2)**
History of child abuse or neglect	229 (<1.0)	171 (2.4)	**401 (1.2)**
Physical fight (two persons, not a brawl)	289 (1.0)	44 (<1.0)	**333 (<1.0)**
Living transition or loss of independent living	253 (<1.0)	34 (<1.0)	**287 (<1.0)**
Traumatic anniversary	168 (<1.0)	81 (1.1)	**249 (<1.0)**
Caregiver burden	170 (<1.0)	42 (<1.0)	**212 (<1.0)**
Caretaker abuse or neglect	25 (<1.0)	26 (<1.0)	**51 (<1.0)**
**Crime and criminal activity**			
Precipitated by another crime	1,113 (4.0)	87 (1.2)	**1,200 (3.5)**
Crime in progress^††^	390 (35.0)	22 (25.3)	**412 (34.3)**
**Suicide and self-harm event**			
History of suicidal thoughts or plans	9,029 (32.7)	2,776 (39.2)	**11,808 (34.0)**
Left a suicide note	7,390 (26.8)	2,493 (35.2)	**9,885 (28.5)**
History of attempting suicide	3,974 (14.4)	2,123 (29.9)	**6,100 (17.6)**
History of nonsuicidal self-harm	604 (2.2)	482 (6.8)	**1,086 (3.1)**
**Suicide disclosure**			
Disclosed suicidal intent	5,874 (21.3)	1,525 (21.5)	**7,400 (21.3)**
Disclosed intent to whom^§§^			
Former or current intimate partner	2,352 (40.0)	542 (35.5)	**2,894 (39.1)**
Other family member	2,134 (36.3)	571 (37.4)	**2,705 (36.6)**
Friend or colleague	821 (14.0)	241 (15.8)	**1,063 (14.4)**
Through social media or other electronic means	319 (5.4)	84 (5.5)	**403 (5.4)**
Health care worker	281 (4.8)	94 (6.2)	**375 (5.1)**
Neighbor	111 (1.9)	33 (2.2)	**144 (1.9)**
Other	507 (8.6)	115 (7.5)	**623 (8.4)**
Unknown	369 (6.3)	108 (7.1)	**477 (6.4)**
**Child decedent incident^¶¶^**			
Previous Child Protective Services report on victim’s household	21 (2.5)	23 (5.9)	**44 (3.6)**
Substance use in decedent’s household	9 (1.1)	5 (1.3)	**14 (1.2)**
Total***	**27,598 (84.1)**	**7,089 (85.5)**	**34,692 (84.4)**

* Includes suicides with one or more precipitating circumstances. More than one circumstance could have been present per decedent.

^†^ Denominator includes suicides with one or more precipitating circumstances. The sums of percentages in columns exceed 100% because more than one circumstance could have been present per decedent.

^§^ Suicide is not reported for decedents aged <10 years because of the small number of suicide deaths per year in this age group.

^¶^ Includes all U.S. states and District of Columbia, with exception of Florida and Hawaii. Data for California are for violent deaths that occurred in 31 counties (Amador, Butte, Colusa, Fresno, Glenn, Humboldt, Imperial, Kings, Lake, Lassen, Los Angeles, Mendocino, Merced, Modoc, Mono, Orange, Placer, Sacramento, San Benito, San Diego, San Francisco, San Luis Obispo, San Mateo, Santa Cruz, Shasta, Siskiyou, Solano, Sonoma, Tehama, Ventura, and Yolo). Data for Texas are for violent deaths that occurred in 13 counties (Bell, Bexar, Collin, Dallas, Denton, El Paso, Fort Bend, Harris, Montgomery, Nueces, Tarrant, Travis, and Williamson).

** Includes decedents with one or more diagnosed current mental health problems; therefore, sums of percentages for the diagnosed conditions exceed 100%. Denominator for the diagnosed conditions include the number of decedents with one or more current diagnosed mental health problems.

^††^ Denominator includes those decedents involved in an incident that was precipitated by another crime.

^§§^ Denominator includes decedents who disclosed intent. The sum of percentages exceeds 100% because more than one response could have been present per decedent.

^¶¶^ Circumstance variables in this category are applicable exclusively to children with a known circumstance. Denominator includes decedents aged 10–17 years (828 males, 389 females, and 1,217 total). Circumstances were unknown for 304 child decedents; total number of children suicide decedents = 1,521.

*** Circumstances were unknown for 6,424 decedents (5,226 males, 1,198 females, and five unknown); total number of suicide decedents = 41,116 (32,824 males, 8,287 females, and five unknown).

The most commonly reported interpersonal or life stressor–related precipitating circumstances for suicide were a recent or impending crisis during the previous or upcoming 2 weeks (30.1%, with the types of crisis listed in Supplementary Table 4, https://stacks.cdc.gov/view/cdc/157365), intimate partner problem (25.0%), physical health problem (19.9%), and argument or conflict (15.7%) (Table 3). Among other circumstances related to suicide, 34.0% of decedents had a history of suicidal thoughts or plans, 28.5% left a suicide note, 21.3% had disclosed suicidal intent to another person, and 17.6% had a history of attempting suicide. Among those who disclosed intent, the greatest proportion of disclosures were to a former or current intimate partner (39.1%), followed by a family member other than an intimate partner (36.6%) and friend or colleague (14.4%).

When examining known circumstances by sex, a larger percentage of female decedents (64.9%) had a current diagnosed mental health problem than did male decedents (44.9%) (Table 3). Female and male suicide decedents had similar percentages of depressed mood at the time of their death (30.2% and 28.9%, respectively). A larger percentage of female decedents (35.7%) than male decedents (19.6%) were known to have been receiving mental health or substance treatment at the time of death. Suicide events including leaving a suicide note, history of suicidal thoughts or plans, history of attempting suicide, and history of nonsuicidal self-harm occurred more frequently among females than males.

Known circumstances were identified in 1,217 (80.0%) suicides of children aged 10–17 years (Table 3). Among child decedents, previous Child Protective Services involvement was more frequently reported in female decedents’ household compared with male decedents’ household (5.9% and 2.5%, respectively). Both male and female child suicide decedents had similar percentages of substance use problems in their household (1.1% and 1.3%, respectively).

### Homicides

#### Sex, Age Group, and Race and Ethnicity

For 2021, a total of 48 NVDRS states (46 states collecting statewide data, 31 California counties, and 13 Texas counties) and the District of Columbia collected data on 21,165 incidents (Supplementary Table 1, https://stacks.cdc.gov/view/cdc/157365) involving 22,288 homicide deaths. The overall homicide rate was 7.9 per 100,000 population (Table 4). The homicide rates were higher among males than females across all age groups, and the rate was highest among adults aged 20–24 years (18.1 per 100,000 population) (Table 4).

**TABLE 4:** Number, percentage,* and rate^†^ of homicides, by selected demographic characteristics of decedent, method of injury used, location in which injury occurred, incident characteristics, and victim-suspect relationship^§^ — National Violent Death Reporting System, 48 states and District of Columbia,^¶^ 2021

Characteristic	Male		Female		Total	
	No. (%)	Rate	No. (%)	Rate	No. (%)	Rate
**Age group, yrs**						
<1	137 (<1.0)	8.8	98 (2.3)	6.6	**236 (1.1)**	**7.7**
1–4	160 (<1.0)	2.4	106 (2.5)	1.7	**266 (1.2)**	**2.0**
5–9	91 (<1.0)	1.0	72 (1.7)	0.9	**163 (<1.0)**	**0.9**
10–14	171 (<1.0)	1.8	76 (1.8)	0.9	**247 (1.1)**	**1.4**
15–19	2,058 (11.4)	21.8	323 (7.6)	3.6	**2,381 (10.7)**	**12.9**
20–24	2,879 (16.0)	30.6	469 (11.0)	5.2	**3,348 (15.0)**	**18.1**
25–29	2,888 (16.0)	29.6	543 (12.7)	5.7	**3,431 (15.4)**	**17.8**
30–34	2,534 (14.1)	25.4	497 (11.6)	5.1	**3,031 (13.6)**	**15.3**
35–44	3,360 (18.7)	18.0	776 (18.2)	4.2	**4,136 (18.6)**	**11.1**
45–54	1,841 (10.2)	10.6	527 (12.3)	3.0	**2,368 (10.6)**	**6.8**
55–64	1,190 (6.6)	6.6	365 (8.5)	2.0	**1,555 (7.0)**	**4.2**
65–74	521 (2.9)	3.9	251 (5.9)	1.7	**772 (3.5)**	**2.7**
75–84	149 (<1.0)	2.5	117 (2.7)	1.5	**266 (1.2)**	**2.0**
≥85	34 (<1.0)	1.9	51 (1.2)	1.6	**85 (<1.0)**	**1.7**
Unknown	2 (<1.0)	—**	0 (0.0)	—	**3 (<1.0)**	**—**
**Race and ethnicity^††^**						
American Indian or Alaska Native	203 (1.1)	18.0	58 (1.4)	5.0	**261 (1.2)**	**11.4**
Asian	164 (<1.0)	2.1	81 (1.9)	1.0	**245 (1.1)**	**1.5**
Black or African American	11,003 (61.1)	62.5	1,857 (43.5)	9.7	**12,860 (57.7)**	**35.0**
Native Hawaiian or other Pacific Islander	24 (<1.0)	11.7	5 (<1.0)	—	**29 (<1.0)**	**7.1**
White	3,484 (19.3)	4.0	1,586 (37.1)	1.8	**5,071 (22.8)**	**2.9**
More than one race	168 (<1.0)	5.2	71 (1.7)	2.2	**239 (1.1)**	**3.7**
Hispanic or Latino	2,885 (16.0)	12.3	591 (13.8)	2.6	**3,476 (15.6)**	**7.5**
Unspecified or unknown	84 (<1.0)	—	22 (<1.0)	—	**107 (<1.0)**	**—**
**Method**						
Firearm	14,942 (82.9)	10.7	2,872 (67.2)	2.0	**17,815 (79.9)**	**6.3**
Sharp instrument	1,322 (7.3)	0.9	493 (11.5)	0.3	**1,815 (8.1)**	**0.6**
Blunt instrument	481 (2.7)	0.3	243 (5.7)	0.2	**724 (3.2)**	**0.3**
Personal weapons (e.g., hands, feet, or fists)	397 (2.2)	0.3	143 (3.3)	0.1	**540 (2.4)**	**0.2**
Hanging, strangulation, or suffocation	138 (<1.0)	0.1	187 (4.4)	0.1	**326 (1.5)**	**0.1**
Motor vehicles (e.g., buses, motorcycles, or other transport vehicles)	99 (<1.0)	<0.1	43 (1.0)	<0.1	**142 (<1.0)**	**<0.1**
Poisoning	56 (<1.0)	<0.1	46 (1.1)	<0.1	**102 (<1.0)**	**<0.1**
Fire or burns	51 (<1.0)	<0.1	29 (<1.0)	<0.1	**80 (<1.0)**	**<0.1**
Fall	28 (<1.0)	<0.1	16 (<1.0)	—	**44 (<1.0)**	**<0.1**
Intentional neglect	15 (<1.0)	—	24 (<1.0)	<0.1	**39 (<1.0)**	**<0.1**
Shaking (e.g., shaken baby syndrome)	24 (<1.0)	<0.1	11 (<1.0)	—	**35 (<1.0)**	**<0.1**
Drowning	16 (<1.0)	—	9 (<1.0)	—	**25 (<1.0)**	**<0.1**
Other (e.g., Taser, electrocution, or nail gun)	33 (<1.0)	—	8 (<1.0)	—	**41 (<1.0)**	**—**
Unknown	413 (2.3)	—	147 (3.4)	—	**560 (2.5)**	**—**
**Location of injury**						
House or apartment	6,458 (35.8)	4.6	2,548 (59.7)	1.8	**9,007 (40.4)**	**3.2**
Street or highway	4,451 (24.7)	3.2	451 (10.6)	0.3	**4,902 (22.0)**	**1.7**
Motor vehicle	2,011 (11.2)	1.4	414 (9.7)	0.3	**2,426 (10.9)**	**0.9**
Parking lot, public garage, or public transport	960 (5.3)	0.7	110 (2.6)	<0.1	**1,070 (4.8)**	**0.4**
Commercial or retail area	922 (5.1)	0.7	102 (2.4)	<0.1	**1,024 (4.6)**	**0.4**
Hotel or motel	279 (1.5)	0.2	86 (2.0)	<0.1	**365 (1.6)**	**0.1**
Natural area	246 (1.4)	0.2	66 (1.5)	<0.1	**312 (1.4)**	**0.1**
Bar or nightclub	276 (1.5)	0.2	25 (<1.0)	<0.1	**301 (1.4)**	**0.1**
Park, playground, sports, or athletic area	248 (1.4)	0.2	40 (<1.0)	<0.1	**288 (1.3)**	**0.1**
Jail or prison	122 (<1.0)	<0.1	2 (<1.0)	—	**124 (<1.0)**	**<0.1**
Other location^§§^	689 (3.8)	—	143 (3.3)	—	**832 (3.7)**	**—**
Unknown	1,353 (7.5)	—	284 (6.6)	—	**1,637 (7.3)**	**—**
**Incident characteristic**						
Emergency medical services present	12,847 (71.3)	9.2	2,862 (67.0)	2.0	**15,710 (70.5)**	**5.5**
Injured at victim’s home	3,360 (18.7)	2.4	1,944 (45.5)	1.4	**5,305 (23.8)**	**1.9**
Child present or witnessed incident	1,295 (7.2)	0.9	660 (15.5)	0.5	**1,955 (8.8)**	**0.7**
Victim was suspected of alcohol use preceding the incident	1,282 (7.1)	0.9	243 (5.7)	0.2	**1,525 (6.8)**	**0.5**
Victim was experiencing homelessness	460 (2.6)	0.3	65 (1.5)	<0.1	**525 (2.4)**	**0.2**
Victim was injured at work or while working	333 (1.8)	0.2	79 (1.8)	<0.1	**412 (1.8)**	**0.2**
Victim was recently released from or admitted to an institutional setting	243 (1.3)	0.2	54 (1.3)	<0.1	**297 (1.3)**	**0.1**
Victim was experiencing housing instability	205 (1.1)	0.2	66 (1.5)	<0.1	**271 (1.2)**	**0.1**
Victim was in public custody when injury occurred	226 (1.3)	0.2	9 (<1.0)	—	**235 (1.1)**	**<0.1**
**Relationship of victim to suspect^¶¶^**						
Acquaintance or friend	1,462 (29.1)	1.0	211 (9.3)	0.2	**1,673 (23.0)**	**0.6**
Spouse or intimate partner (current or former)	374 (7.5)	0.3	1,155 (51.0)	0.8	**1,529 (21.0)**	**0.5**
Other person, known to victim	1,150 (22.9)	0.8	201 (8.9)	0.1	**1,351 (18.5)**	**0.5**
Stranger	910 (18.1)	0.7	157 (6.9)	0.1	**1,067 (14.6)**	**0.4**
Other relative	403 (8.0)	0.3	147 (6.5)	0.1	**550 (7.6)**	**0.2**
Parent***	251 (5.0)	0.2	181 (8.0)	0.1	**432 (5.9)**	**0.2**
Child***	251 (5.0)	0.2	170 (7.5)	0.1	**422 (5.8)**	**0.2**
Child of suspect’s boyfriend or girlfriend (e.g., child killed by mom's boyfriend)	65 (1.3)	<0.1	33 (1.5)	<0.1	**98 (1.3)**	**<0.1**
Rival gang member	78 (1.6)	<0.1	3 (<1.0)	—	**81 (1.1)**	**<0.1**
Other relationship^†††^	74 (1.5)	—	7 (<1.0)	—	**81 (1.1)**	**—**
**Total**	**18,015 (100)**	**12.8**	**4,271 (100)**	**3.0**	**22,288 (100)**	**7.9**

* Percentages might not total 100% due to rounding. Sex was unknown for two decedents.

^†^ Per 100,000 population.

^§^ The following sentence can be used as a guide for interpreting the victim-suspect relationship: “The victim is the [insert relationship] of the suspect.” For example, when a parent kills a child, the relationship is “child” not “parent” (“The victim is the child of the suspect”). This sentence is intended to be a general guide; certain relationships might not be captured by this sentence (e.g., other person known to victim or victim was law enforcement officer killed in the line of duty).

^¶^ Includes all U.S. states and District of Columbia, with exception of Florida and Hawaii. Data for California are for violent deaths that occurred in 31 counties (Amador, Butte, Colusa, Fresno, Glenn, Humboldt, Imperial, Kings, Lake, Lassen, Los Angeles, Mendocino, Merced, Modoc, Mono, Orange, Placer, Sacramento, San Benito, San Diego, San Francisco, San Luis Obispo, San Mateo, Santa Cruz, Shasta, Siskiyou, Solano, Sonoma, Tehama, Ventura, and Yolo). Data for Texas are for violent deaths that occurred in 13 counties (Bell, Bexar, Collin, Dallas, Denton, El Paso, Fort Bend, Harris, Montgomery, Nueces, Tarrant, Travis, and Williamson). Denominators for the rates for California and Texas represent only the populations of the counties from which the data were collected.

** Dashes indicate cell data are suppressed because number of decedents is <20 or when characteristic response is “Other” or “Unknown.”

^††^ Persons of Hispanic or Latino (Hispanic) origin might be of any race but are categorized as Hispanic; all racial groups are non-Hispanic.

^§§^ Other location includes (in descending order): abandoned house, building, or warehouse; supervised residential facility; industrial or construction area; office building; preschool, school, college, or school bus; hospital or medical facility; synagogue, church, or temple; farm; railroad tracks; cemetery, graveyard, or other burial ground; bridge; and other unspecified location.

^¶¶^ Percentage is based on the number of homicide decedents with a known victim-suspect relationship (n = 7,284 [32.7%]; 5,018 [27.9%] males and 2,265 [53.0%] females); the victim-suspect relationship was unknown for 15,004 decedents.

*** Includes adoptive family members (e.g., adopted child), stepfamily members (e.g., stepparent), and foster family members (e.g., foster child).

^†††^ Other victim-suspect relationship includes (in descending order): intimate partner of suspect’s parent (e.g., teenager kills his mother’s boyfriend) and victim was law enforcement officer injured in line of duty.

The homicide rate for men aged 20–24 years (30.6 per 100,000 population) was about six times the rate for females in the same age group (5.2 per 100,000 population). Among males, the rate of homicide was highest among adults aged 20–24 years (30.6 per 100,000 population) and 25–29 years (29.6 per 100,000 population). Among females, the rate of homicide was highest among infants (i.e., children aged <1 year; 6.6 per 100,000 population). Among all children who were homicide victims, the overall homicide rate for infants (7.7 per 100,000 population) was 3.9 times the overall rate for children aged 1–4 years (2.0 per 100,000 population) and 8.6 times the rate for children aged 5–9 years (0.9 per 100,000 population).

Black persons accounted for 61.1% of male homicide victims and 43.5% of female homicide victims (Table 4). Black males had the highest rate of homicide compared with males in all other racial and ethnic groups (62.5 per 100,000 population); this rate was 29.8 times the rate for Asian males (2.1 per 100,000 population), 15.6 times the rate for White males (4.0 per 100,000 population), 5.3 times the rate for NH/PI males (11.7 per 100,000 population), 5.1 times the rate for Hispanic males (12.3 per 100,000 population), 3.5 times the rate for AI/AN males (18.0 per 100,000 population), and 12.0 times the rate for males with more than one race (5.2 per 100,000 population). Among females, the homicide rate was also highest among Black females (9.7 per 100,000 population) (Table 4), followed by AI/AN females (5.0 per 100,000 population), Hispanic females (2.6 per 100,000 population), and females with more than one race (2.2 per 100,000 population), White females (1.8 per 100,000 population), and Asian females (1.0 per 100,000 population).

#### Method and Location of Injury 

The weapons most commonly used in homicides were firearms, used in 79.9% of homicides overall, followed by a sharp instrument (8.1%); a blunt instrument (3.2%); personal weapons (e.g., hands, feet, or fists; 2.4%); and hanging, strangulation, or suffocation (1.5%) (Table 4). The method was unknown in 2.5% of homicides. A firearm was the most common method of injury for both males (82.9%) and females (67.2%); however, the firearm homicide rate for males (10.7 per 100,000 population) was 5.4 times the rate for females (2.0 per 100,000 population). A larger proportion of homicides among females than males involved a sharp instrument (11.5% versus 7.3%, respectively); a blunt instrument (5.7% versus 2.7%, respectively); hanging, strangulation, or suffocation (4.4% versus <1.0%, respectively); and personal weapons (3.3% and 2.2%, respectively). Among all homicide victims, a house or apartment was the most common location of homicide (40.4%), followed by a street or highway (22.0%); a motor vehicle (10.9%); and a parking lot, public garage, or public transport (4.8%). A larger proportion of homicides among females (59.7%) than among males (35.8%) occurred at a house or apartment, whereas a larger proportion of homicides among males (24.7%) than among females (10.6%) occurred on a street or highway.

#### Incident Characteristics

Emergency medical services were present for a large percentage of homicide victims (70.5%) (Table 4). A larger proportion of homicides among females than males occurred at the victim’s home (45.5% and 18.7%, respectively) and involved a child who was present or witnessed the incident (15.5% and 7.2%, respectively). Among all homicide victims, 6.8% victims were suspected of alcohol use preceding the incident. A small proportion of all homicide victims were experiencing homelessness (2.4%), housing instability (1.2%), or were recently injured at work or while working (1.8%).

#### Victim-Suspect Relationship

The relationship of the victim to the suspect was known for 32.7% of homicides (27.9% of males and 53.0% of females) (Table 4). For males, when the relationship was known, the victim-suspect relationship was most often an acquaintance or friend (29.1%); other person known to the victim, but the exact nature of the relationship was unclear (22.9%); a stranger (18.1%); relative other than a parent or child (8.0%); or a current or former intimate partner (7.5%). For females, when the relationship was known, approximately half (51.0%) of suspects were a current or former intimate partner, followed by an acquaintance or friend (9.3%); other person known to victim, but the exact nature of the relationship was unclear (8.9%); a parent (8.0%); a child (7.5%); or a stranger (6.9%).

#### Precipitating Circumstances

Precipitating circumstances were identified in 70.3% of homicides (Table 5). One third of homicides with known circumstances were precipitated by an argument or conflict (33.5%), and 14.3% of homicides with known circumstances were related to intimate partner violence (Table 5). Intimate partner violence–related deaths include deaths related to conflict or violence between current or former intimate partners and also include deaths associated with intimate partner violence that are not deaths of the intimate partners themselves (e.g., a former boyfriend killing an ex-partner’s new boyfriend). Homicides also were commonly precipitated by another crime (21.9%); in 67.0% of those cases, the crime was in progress at the time of the incident. The most frequent types of precipitating crimes were assault or homicide (45.5%), robbery (29.6%), burglary (11.6%), drug trade^§§^ (10.1%), motor vehicle theft (4.5%), rape or sexual assault (2.1%), and arson (1.1%) (Supplementary Table 5, https://stacks.cdc.gov/view/cdc/157365). A physical fight between two persons (14.3%), a drive-by shooting (12.6%), and drug involvement (e.g., relating to drug use or illegal drug trafficking; 9.1%) were other common precipitating circumstances. A recent or impending crisis during the previous or upcoming 2 weeks was present for 7.6% of decedents (type of crisis listed in Supplementary Table 6, https://stacks.cdc.gov/view/cdc/157365). Toxicology results in homicide deaths are available (Supplementary Table 7, https://stacks.cdc.gov/view/cdc/157365).

**TABLE 5:** Number* and percentage^†^ of homicides, by decedent’s sex and precipitating circumstances — National Violent Death Reporting System, 48 states and District of Columbia,^§^ 2021

Precipitating circumstance	Male	Female	Total
	No. (%)	No. (%)	No. (%)
**Mental health and substance use**			
Substance use problem (excludes alcohol)	1,756 (14.1)	347 (10.7)	**2,103 (13.4)**
Current diagnosed mental health problem	583 (4.7)	263 (8.1)	**846 (5.4)**
Alcohol problem	480 (3.9)	117 (3.6)	**597 (3.8)**
History of ever being treated for a mental health or substance use problem	320 (2.6)	156 (4.8)	**476 (3.0)**
Current mental health treatment	176 (1.4)	93 (2.9)	**269 (1.7)**
Non-adherence to mental health or substance use treatment	50 (<1.0)	22 (<1.0)	**72 (<1.0)**
Current depressed mood	34 (<1.0)	18 (<1.0)	**52 (<1.0)**
Other addiction (e.g., gambling or sex)	28 (<1.0)	7 (<1.0)	**35 (<1.0)**
**Interpersonal factor**			
Intimate partner violence–related	894 (7.2)	1,348 (41.5)	**2,242 (14.3)**
Other relationship problem (nonintimate and nonfamily)	1,029 (8.3)	168 (5.2)	**1,197 (7.6)**
Family relationship problem	620 (5.0)	269 (8.3)	**889 (5.7)**
Jealousy (lovers’ triangle)	240 (1.9)	86 (2.6)	**326 (2.1)**
Victim of interpersonal violence during past month	107 (<1.0)	137 (4.2)	**244 (1.6)**
Perpetrator of interpersonal violence during past month	184 (1.5)	18 (<1.0)	**202 (1.3)**
**Life stressor**			
Argument or conflict	4,244 (34.2)	1,012 (31.2)	**5,256 (33.5)**
Physical fight (two persons, not a brawl)	1,924 (15.5)	314 (9.7)	**2,238 (14.3)**
Crisis during previous or upcoming 2 weeks	884 (7.1)	305 (9.4)	**1,189 (7.6)**
Household had contact with local authorities	315 (2.5)	245 (7.5)	**560 (3.6)**
Family stressor	103 (<1.0)	89 (2.7)	**192 (1.2)**
History of child abuse or neglect	76 (<1.0)	56 (1.7)	**132 (<1.0)**
Living transition or loss of independent living	13 (<1.0)	7 (<1.0)	**20 (<1.0)**
**Crime and criminal activity**			
Precipitated by another crime	2,840 (22.9)	589 (18.1)	**3,430 (21.9)**
Crime in progress^¶^	1,931 (68.0)	366 (62.1)	**2,297 (67.0)**
Drug involvement	1,249 (10.1)	169 (5.2)	**1,419 (9.1)**
Gang related	930 (7.5)	79 (2.4)	**1,009 (6.4)**
**Homicide event**			
Drive-by shooting	1,728 (13.9)	245 (7.5)	**1,973 (12.6)**
Victim used a weapon	1,150 (9.3)	61 (1.9)	**1,211 (7.7)**
Walk-by assault	1,031 (8.3)	113 (3.5)	**1,144 (7.3)**
Caretaker abuse or neglect led to death	422 (3.4)	329 (10.1)	**752 (4.8)**
Justifiable self-defense	467 (3.8)	18 (<1.0)	**485 (3.1)**
Random violence	392 (3.2)	87 (2.7)	**479 (3.1)**
Mentally ill suspect	244 (2.0)	184 (5.7)	**428 (2.7)**
Victim was a bystander	202 (1.6)	141 (4.3)	**343 (2.2)**
Brawl	254 (2.0)	19 (<1.0)	**273 (1.7)**
Victim was an intervener assisting a crime victim	135 (1.1)	28 (<1.0)	**163 (1.0)**
Stalking	36 (<1.0)	40 (1.2)	**76 (<1.0)**
Prostitution	44 (<1.0)	21 (<1.0)	**65 (<1.0)**
Victim was a police officer on duty	40 (<1.0)	5 (<1.0)	**45 (<1.0)**
Mercy killing	7 (<1.0)	7 (<1.0)	**14 (<1.0)**
Hate crime	9 (<1.0)	3 (<1.0)	**12 (<1.0)**
Child victim incident**			
Previous Child Protective Services report on victim’s household	62 (6.2)	35 (9.5)	**97 (7.1)**
Substance use in victim’s household	42 (4.2)	35 (9.5)	**77 (5.6)**
Corporal punishment	10 (<1.0)	9 (<1.0)	**19 (<1.0)**
**Total^††^**	**12,426 (69.0)**	**3,246 (76.0)**	**15,674 (70.3)**

* Includes homicides with one or more precipitating circumstances. Total numbers do not equal the sums of the columns because more than one circumstance could have been present per decedent.

^†^ Denominator includes those homicides with one or more precipitating circumstances. The sums of percentages in columns exceed 100% because more than one circumstance could have been present per decedent.

^§^ Includes all U.S. states and District of Columbia, with exception of Florida and Hawaii. Data for California are for violent deaths that occurred in 31 counties (Amador, Butte, Colusa, Fresno, Glenn, Humboldt, Imperial, Kings, Lake, Lassen, Los Angeles, Mendocino, Merced, Modoc, Mono, Orange, Placer, Sacramento, San Benito, San Diego, San Francisco, San Luis Obispo, San Mateo, Santa Cruz, Shasta, Siskiyou, Solano, Sonoma, Tehama, Ventura, and Yolo). Data for Texas are for violent deaths that occurred in 13 counties (Bell, Bexar, Collin, Dallas, Denton, El Paso, Fort Bend, Harris, Montgomery, Nueces, Tarrant, Travis, and Williamson).

^¶^ Denominator includes those decedents involved in an incident that was precipitated by another crime.

** Circumstance variables in this category are applicable exclusively to children. Denominator includes decedents aged 0–17 years with known circumstance (1,002 males, 369 females, and 1,372 total). Circumstances were unknown for 528 child decedents; total number of children homicide decedents = 1,900.

^††^ Circumstances were unknown for 6,614 decedents (5,589 males and 1,025 females); total number of homicide decedents = 22,288 (18,015 males and 4,271 females).

Among the identified homicide circumstances, multiple differences were noted by decedent’s sex, and intimate partner violence accounted for the largest percentage difference. Intimate partner violence was a precipitating circumstance for approximately 41.5% of homicides among females but only 7.2% of homicides among males (Table 5). In incidents where intimate partner violence was a precipitating circumstance and victim-suspect relationship was known, the suspect was a current or former intimate partner in 93.5% of homicides among females and 49.8% of homicides among males. Females were more often the direct victims of intimate partner violence–related homicides, whereas males were more often corollary victims. A larger proportion of homicides of females than males also resulted from caregiver abuse or neglect (10.1% versus 3.4%) or were perpetrated by a suspect with a mental health problem (e.g., schizophrenia or other psychotic conditions, depression, or posttraumatic stress disorder) (5.7% versus 2.0%) (Table 5). A larger proportion of homicides of males than females were preceded by a physical fight (15.5% versus 9.7%), involved drugs (10.1% versus 5.2%), or were gang related (7.5% versus 2.4%). A larger proportion of male homicide victims (9.3%) than female homicide victims (1.9%) also were reported to have used a weapon during the incident.

Known circumstances of child victim incidents were identified in 1,372 (72.2%) homicides of children aged <18 years (Table 5). A larger proportion of female victims’ households had previous Child Protective Services reports (9.5% versus 6.2%) or reported substance use problems in their households (9.5% versus 4.2%) compared with male victims’ households.

### Legal Intervention Deaths

#### Sex, Age Group, and Race and Ethnicity

For 2021, a total of 48 NVDRS states (46 states collecting statewide data, 31 California counties, and 13 Texas counties) and the District of Columbia collected data on 912 incidents involving 923 legal intervention deaths (Supplementary Table 1, https://stacks.cdc.gov/view/cdc/157365). The highest rate of legal intervention death by age group was among men aged 30–34 years (1.7 per 100,000 population), followed by men aged 25–29 years (1.5 per 100,000 population) and 35–44 years (1.3 per 100,000 population) (Table 6). Nearly all legal intervention deaths were among males (94.5%). Although White males accounted for 43.8% of all legal intervention deaths, AI/AN males had the highest legal intervention death rate (1.9 per 100,000 population), representing a rate 3.8 times that of White males (0.5 per 100,000 population). The legal intervention death rate for Black males (1.4 per 100,000 population) was 2.8 times the rate for White males. The legal intervention death rate for Hispanic males was 0.7 per 100,000 population.

**TABLE 6:** Number, percentage,* and rate^†^ of legal intervention^§^ deaths, by selected demographic characteristics of decedent, method used, location where injury occurred, and incident characteristics — National Violent Death Reporting System, 48 states and District of Columbia,^¶^ 2021

Characteristic	Male		Female		Total	
	No. (%)	Rate	No. (%)	Rate	No. (%)	Rate
**Age group, yrs**						
<10	0 (—)	—**	0 (—)	—	**0 (—)**	**—**
10–14	2 (<1.0)	—	1 (2.0)	—	**3 (<1.0)**	**—**
15–19	29 (3.3)	0.3	6 (11.8)	—	**35 (3.8)**	**0.2**
20–24	89 (10.2)	1.0	1 (2.0)	—	**90 (9.8)**	**0.5**
25–29	142 (16.3)	1.5	10 (19.6)	—	**152 (16.5)**	**0.8**
30–34	168 (19.3)	1.7	9 (17.6)	—	**177 (19.2)**	**0.9**
35–44	238 (27.3)	1.3	11 (21.6)	—	**249 (27.0)**	**0.7**
45–54	116 (13.3)	0.7	11 (21.6)	—	**127 (13.8)**	**0.4**
55–64	69 (7.9)	0.4	0 (—)	—	**69 (7.5)**	**0.2**
65–74	12 (1.4)	—	2 (3.9)	—	**14 (1.5)**	**—**
75–84	6 (<1.0)	—	0 (—)	—	**6 (<1.0)**	**—**
≥85	1 (<1.0)	—	0 (—)	—	**1 (<1.0)**	**—**
**Race and ethnicity^††^**						
American Indian or Alaska Native	21 (2.4)	1.9	0 (—)	—	**21 (2.3)**	**0.9**
Asian American	12 (1.4)	—	0 (—)	—	**12 (1.3)**	**—**
Black or African American	247 (28.3)	1.4	9 (17.6)	—	**256 (27.7)**	**0.7**
Native Hawaiian or other Pacific Islander	0 (—)	—	0 (—)	—	**0 (—)**	**—**
White	404 (46.3)	0.5	35 (68.6)	<0.1	**439 (47.6)**	**0.3**
More than one race	16 (1.8)	—	0 (—)	—	**16 (1.7)**	**—**
Hispanic or Latino	170 (19.5)	0.7	7 (13.7)	—	**177 (19.2)**	**0.4**
Unspecified or unknown	2 (<1.0)	—	0 (—)	—	**2 (<1.0)**	**—**
**Method of injury**						
Firearm	760 (87.2)	0.5	41 (80.4)	<0.1	**801 (86.8)**	**0.3**
Motor vehicles (e.g., buses, motorcycles, and other transport vehicles)	39 (4.5)	<0.1	5 (9.8)	—	**44 (4.8)**	**<0.1**
Drowning	8 (<1.0)	—	0 (—)	—	**8 (<1.0)**	**—**
Fire or burns	5 (<1.0)	—	1 (2.0)	—	**6 (<1.0)**	**—**
Blunt instrument	4 (<1.0)	—	0 (—)	—	**4 (<1.0)**	**—**
Poisoning	3 (<1.0)	—	0 (—)	—	**3 (<1.0)**	**—**
Personal weapons (e.g., hands, feet, or fists)	3 (<1.0)	—	0 (—)	—	**3 (<1.0)**	**—**
Hanging, strangulation, or suffocation	2 (<1.0)	—	0 (—)	—	**2 (<1.0)**	**—**
Fall	2 (<1.0)	—	0 (—)	—	**2 (<1.0)**	**—**
Other (e.g., Taser, electrocution, or nail gun)	9 (1.0)	—	0 (—)	—	**9 (<1.0)**	**—**
Unknown	37 (4.2)	—	4 (7.8)	—	**41 (4.4)**	**—**
**Location of injury**						
House or apartment	297 (34.1)	0.2	15 (29.4)	—	**312 (33.8)**	**0.1**
Street or highway	229 (26.3)	0.2	12 (23.5)	—	**241 (26.1)**	**<0.1**
Motor vehicle	85 (9.7)	<0.1	12 (23.5)	—	**97 (10.5)**	**<0.1**
Parking lot, public garage, or public transport	53 (6.1)	<0.1	0 (—)	—	**53 (5.7)**	**<0.1**
Commercial or retail area	40 (4.6)	<0.1	4 (7.8)	—	**44 (4.8)**	**<0.1**
Natural area	33 (3.8)	<0.1	0 (—)	—	**33 (3.6)**	**<0.1**
Other location^§§^	79 (9.1)	—	5 (9.8)	—	**84 (9.1)**	**—**
Unknown	56 (6.4)	—	3 (5.9)	—	**59 (6.4)**	**—**
**Incident characteristic**						
Emergency medical services present	688 (78.9)	0.5	42 (82.4)	<0.1	**730 (79.1)**	**0.3**
Victim was in public custody when injury occurred	259 (29.7)	0.2	14 (27.5)	—	**273 (29.6)**	**0.1**
Injured at victim’s home	214 (24.5)	0.2	13 (25.5)	—	**227 (24.6)**	**<0.1**
Victim was suspected of alcohol use preceding the incident	105 (12.0)	<0.1	3 (5.9)	—	**108 (11.7)**	**<0.1**
Child present or witnessed incident	47 (5.4)	<0.1	5 (9.8)	—	**52 (5.6)**	**<0.1**
Victim was recently released from or admitted to an institutional setting	38 (4.4)	<0.1	3 (5.9)	—	**41 (4.4)**	**<0.1**
Victim was experiencing homelessness	26 (3.0)	<0.1	1 (2.0)	—	**27 (2.9)**	**<0.1**
Victim was experiencing housing instability	23 (2.6)	<0.1	1 (2.0)	—	**24 (2.6)**	**<0.1**
Victim was injured at work or while working	4 (<1.0)	—	1 (2.0)	—	**5 (<1.0)**	**—**
**Total**	**872 (100)**	**0.6**	**51 (100)**	**<0.1**	**923 (100)**	**0.3**

* Percentages might not total 100% due to rounding.

^†^ Per 100,000 population.

^§^ The term “legal intervention” does not denote the lawfulness or legality of the circumstances surrounding the death.

^¶^ Includes all U.S. states and District of Columbia, with exception of Florida and Hawaii. Data for California are for violent deaths that occurred in 31 counties (Amador, Butte, Colusa, Fresno, Glenn, Humboldt, Imperial, Kings, Lake, Lassen, Los Angeles, Mendocino, Merced, Modoc, Mono, Orange, Placer, Sacramento, San Benito, San Diego, San Francisco, San Luis Obispo, San Mateo, Santa Cruz, Shasta, Siskiyou, Solano, Sonoma, Tehama, Ventura, and Yolo). Data for Texas are for violent deaths that occurred in 13 counties (Bell, Bexar, Collin, Dallas, Denton, El Paso, Fort Bend, Harris, Montgomery, Nueces, Tarrant, Travis, and Williamson). Denominators for the rates for California and Texas represent only the populations of the counties from which the data were collected.

** Dashes indicate cell data are suppressed because number of decedents is <20 or when characteristic response is “Other” or “Unknown.”

^††^ Persons of Hispanic or Latino (Hispanic) origin might be of any race but are categorized as Hispanic; all racial groups are non-Hispanic.

^§§^ Other location includes (in descending order): hotel or motel; park, playground, or public use area; sports or athletic area; jail or prison; hospital or medical facility; bar or nightclub; industrial or construction area; office building; cemetery, graveyard, or other burial ground; preschool, school, college, or school bus; supervised residential facility; bridge; and other unspecified location.

#### Method and Location of Injury

A firearm was used in a majority (86.8%) of legal intervention deaths (Table 6). Legal intervention deaths occurred most frequently in a house or apartment (33.8%), followed by a street or highway (26.1%) or a motor vehicle (10.5%).

#### Incident Characteristics

Among all legal intervention deaths, emergency medical services were present for a large percentage of deaths (79.1%), and more than one quarter of decedents were in public custody when the injury occurred (29.6%) (Table 6). Approximately one quarter of legal intervention deaths occurred at the decedent’s own home (24.6%); 11.7% of decedents were suspected of alcohol use preceding the incident, and 5.6% of decedents had a child present or witnessing the incident. A small proportion of legal intervention deaths involved decedents experiencing housing instability (2.6%) or homelessness (2.9%) at the time of death.

#### Precipitating Circumstances

Precipitating circumstances were identified in 92.1% of legal intervention deaths (Table 7). The decedent reportedly used a weapon in 66.8% of legal intervention death cases. In 24.9% of legal intervention deaths with known circumstances, a substance use problem (other than alcohol) was reported as a contributing factor, and 19.9% of decedents reportedly had a current diagnosed mental health problem. An argument or conflict or physical fight precipitated 14.0% and 9.8% of legal intervention deaths, respectively. A recent or impending crisis during the previous or upcoming 2 weeks was reported in 11.5% of legal intervention deaths (types of crises listed in Supplementary Table 8, https://stacks.cdc.gov/view/cdc/157365). Among legal intervention deaths with known circumstances, being a perpetrator of interpersonal violence during the past month (11.9%), intimate partner violence (6.9%), family relationship problems (6.9%), and drug involvement (4.2%) were other notable precipitating circumstances. Toxicology results in deaths of legal intervention deaths are available (Supplementary Table 9, https://stacks.cdc.gov/view/cdc/157365).

**TABLE 7:** Number* and percentage^†^ of legal intervention^§^ deaths, by decedent’s sex and precipitating circumstances — National Violent Death Reporting System, 48 states and District of Columbia,^¶^ 2021

Precipitating circumstance	Male	Female	Total
	No. (%)	No. (%)	No. (%)
**Mental health and substance use**			
Substance use problem (excludes alcohol)	193 (24.0)	19 (41.3)	212 (24.9)
Current diagnosed mental health problem	152 (18.9)	17 (37.0)	169 (19.9)
History of ever being treated for a mental health problem	92 (11.4)	10 (21.7)	102 (12.0)
Alcohol problem	71 (8.8)	4 (8.7)	75 (8.8)
Current mental health treatment	51 (6.3)	6 (13.0)	57 (6.7)
Current depressed mood	32 (4.0)	1 (2.2)	33 (3.9)
Nonadherence to mental health or substance use treatment	30 (3.7)	2 (4.3)	32 (3.8)
Other addiction (e.g., gambling or sex)	7 (<1.0)	0 (—)	7 (<1.0)
**Interpersonal factor**			
Perpetrator of interpersonal violence during past month	98 (12.2)	3 (6.5)	101 (11.9)
Intimate partner violence–related	59 (7.3)	0 (0.0)	59 (6.9)
Family relationship problem	57 (7.1)	2 (4.3)	59 (6.9)
Other relationship problem (nonintimate and nonfamily)	10 (1.2)	0 (—)	10 (1.2)
Victim of interpersonal violence during past month	2 (<1.0)	0 (—)	2 (<1.0)
Jealousy (lovers’ triangle)	2 (<1.0)	0 (—)	2 (<1.0)
**Life stressor**			
Argument or conflict	115 (14.3)	4 (8.7)	119 (14.0)
Crisis during previous or upcoming 2 weeks	96 (11.9)	2 (4.3)	98 (11.5)
Physical fight (two persons, not a brawl)	80 (10.0)	3 (6.5)	83 (9.8)
Family stressor	13 (1.6)	1 (2.2)	14 (1.6)
History of child abuse or neglect	2 (<1.0)	0 (—)	2 (<1.0)
**Crime and criminal activity**			
Drug involvement	34 (4.2)	2 (4.3)	36 (4.2)
Gang related	4 (<1.0)	0 (—)	4 (<1.0)
**Homicide event**			
Victim used a weapon	541 (67.3)	27 (58.7)	568 (66.8)
Brawl	6 (<1.0)	1 (2.2)	7 (<1.0)
Caretaker abuse or neglect led to death	5 (<1.0)	0 (—)	5 (<1.0)
Victim was a bystander	0 (—)	3 (6.5)	3 (<1.0)
Random violence	2 (<1.0)	1 (2.2)	3 (<1.0)
Stalking	3 (<1.0)	0 (—)	3 (<1.0)
Victim was an intervener assisting a crime victim	2 (<1.0)	0 (—)	2 (<1.0)
Total**	**804 (92.2)**	**46 (90.2)**	**850 (92.1)**

* Includes deaths with one or more precipitating circumstances. Total numbers do not equal the sums of the columns because more than one circumstance could have been present per decedent.

^†^ Denominator includes those deaths with one or more precipitating circumstances. The sums of percentages in columns exceed 100% because more than one circumstance could have been present per decedent.

^§^ The term “legal intervention” does not denote the lawfulness or legality of the circumstances surrounding the death.

^¶^ Includes all U.S. states and District of Columbia, with exception of Florida and Hawaii. Data for California are for violent deaths that occurred in 31 counties (Amador, Butte, Colusa, Fresno, Glenn, Humboldt, Imperial, Kings, Lake, Lassen, Los Angeles, Mendocino, Merced, Modoc, Mono, Orange, Placer, Sacramento, San Benito, San Diego, San Francisco, San Luis Obispo, San Mateo, Santa Cruz, Shasta, Siskiyou, Solano, Sonoma, Tehama, Ventura, and Yolo). Data for Texas are for violent deaths that occurred in 13 counties (Bell, Bexar, Collin, Dallas, Denton, El Paso, Fort Bend, Harris, Montgomery, Nueces, Tarrant, Travis, and Williamson).

** Circumstances were unknown for 73 decedents (68 males and five females); total number of legal intervention deaths = 923 (872 males and 51 females).

### Unintentional Firearm Injury Deaths

#### Sex, Age Group, and Race and Ethnicity

In 2021, a total of 48 NVDRS states (46 states collecting statewide data, 31 California counties, and 13 Texas counties) and the District of Columbia collected data on 555 incidents involving 555 unintentional firearm injury deaths (Supplementary Table 1, https://stacks.cdc.gov/view/cdc/157365). Nearly half (n = 239; 43.1%) of these deaths were self-inflicted, and 202 deaths (36.4%) were known to be inflicted by another person; for the remaining 114 deaths (20.5%), it was unknown whether the injury was self- or other-inflicted. Males accounted for 84.3% of decedents (Table 8). Persons aged ≤24 years accounted for more than half (56.0%) of all unintentional firearm injury deaths. Approximately half of decedents were White persons (49.5%), followed by Black persons (34.8%).

**TABLE 8:** Number and percentage* of unintentional firearm injury deaths, by selected demographic characteristics of decedent, location of injury, type of firearm, and incident characteristics — National Violent Death Reporting System, 48 states and District of Columbia,^†^ 2021

Characteristic	No. (%)
**Sex**	
Male	468 (84.3)
Female	87 (15.7)
**Race and ethnicity^§^**	
American Indian or Alaska Native	7 (1.3)
Asian	8 (1.4)
Black or African American	193 (34.8)
Native Hawaiian or other Pacific Islander	0 (—)
White	275 (49.5)
More than one race	10 (1.8)
Hispanic or Latino	59 (10.6)
Unspecified or unknown	3 (<1.0)
**Age group, yrs**	
<1	1 (<1.0)
1–4	58 (10.5)
5–9	38 (6.8)
10–14	39 (7.0)
15–19	95 (17.1)
20–24	80 (14.4)
25–29	35 (6.3)
30–34	34 (6.1)
35–44	64 (11.5)
45–54	32 (5.8)
55–64	35 (6.3)
65–74	28 (5.0)
75–84	13 (2.3)
≥85	3 (<1.0)
**Location of injury**	
House or apartment	407 (73.3)
Motor vehicle	32 (5.8)
Natural area	23 (4.1)
Street or highway	11 (2.0)
Commercial or retail area	8 (1.4)
Parking lot, public garage, or public transport	7 (1.3)
Hotel or motel	4 (<1.0)
Other location^¶^	24 (4.3)
Unknown	39 (7.0)
**Firearm type**	
Handgun	341 (61.4)
Rifle	55 (9.9)
Shotgun	27 (4.9)
Other	5 (<1.0)
Unknown	127 (22.9)
**Incident characteristic**	
Emergency medical services present	406 (73.2)
Injured at victim’s home	273 (49.2)
Child present or witnessed incident	152 (27.4)
Victim was suspected of alcohol use preceding the incident	72 (13.0)
Victim was experiencing housing instability	4 (<1.0)
Victim was injured at work or while working	4 (<1.0)
Victim was in public custody when injury occurred	4 (<1.0)
Victim was experiencing homelessness	2 (<1.0)
Victim was recently released from or admitted to an institutional setting	1 (<1.0)
**Total**	**555 (100)**

* Percentages might not total 100% due to rounding.

^†^ Includes all U.S. states and District of Columbia, with exception of Florida and Hawaii. Data for California are for violent deaths that occurred in 31 counties (Amador, Butte, Colusa, Fresno, Glenn, Humboldt, Imperial, Kings, Lake, Lassen, Los Angeles, Mendocino, Merced, Modoc, Mono, Orange, Placer, Sacramento, San Benito, San Diego, San Francisco, San Luis Obispo, San Mateo, Santa Cruz, Shasta, Siskiyou, Solano, Sonoma, Tehama, Ventura, and Yolo). Data for Texas are for violent deaths that occurred in 13 counties (Bell, Bexar, Collin, Dallas, Denton, El Paso, Fort Bend, Harris, Montgomery, Nueces, Tarrant, Travis, and Williamson).

^§^ Persons of Hispanic or Latino (Hispanic) origin might be of any race but are categorized as Hispanic; all racial groups are non-Hispanic.

^¶^ Other location includes (in descending order) park, playground, or public use area; sports or athletic area; bar or nightclub; industrial or construction area; farm; abandoned house, building, or warehouse; synagogue, church, or temple; hospital or medical facility; and other unspecified location.

#### Location of Injury and Firearm Type

Among unintentional firearm injury deaths, 73.3% occurred in a house or apartment, followed by a motor vehicle (5.8%) or a natural area (4.1%) (Table 8). The majority of unintentional firearm injury deaths involved a handgun (61.4%), followed by a rifle (9.9%) or a shotgun (4.9%). The firearm type was unknown in approximately one quarter (22.9%) of unintentional firearm injury deaths.

#### Incident Characteristics

Among unintentional firearm injury deaths, emergency medical services were present for most of deaths (73.2%) (Table 8). Approximately half of all unintentional firearm injury deaths occurred at the decedent's own home (49.2%), and a child was present or witnessed the incident in 27.4% of unintentional firearm injury deaths. Furthermore, 13.0% of the decedents were suspected of alcohol use preceding the incident.

#### Context and Circumstances of Injury

The context and circumstances of injury were identified in 83.6% of unintentional firearm injury deaths (Table 9). Among those with context and circumstance information, the context of injury for nearly one half (44.0%) of unintentional firearm injury deaths was playing with a firearm. Other contexts of injury were showing the firearm to others (15.1%), hunting (5.4%), and loading or unloading the firearm (5.2%). Approximately one fourth (23.7%) of unintentional firearm injury deaths were precipitated by a person unintentionally pulling the trigger; 20.1% resulted from a person mistakenly thinking the firearm was unloaded, with 9.5% due to a disengaged magazine and 10.6% occurring when the magazine was engaged; and 7.8% of deaths were because of the firearm being mistaken for a toy.

**TABLE 9:** Number and percentage* of unintentional firearm injury deaths, by context and circumstances of injury — National Violent Death Reporting System, 48 states and District of Columbia,^†^ 2021

Characteristic	No. (%)
**Context of injury**	
Playing with gun	204 (44.0)
Showing gun to others	70 (15.1)
Hunting	25 (5.4)
Loading or unloading gun	24 (5.2)
Cleaning gun	19 (4.1)
Target shooting	8 (1.7)
Celebratory firing	2 (<1.0)
Other context of injury	86 (18.5)
**Circumstance of injury**	
Unintentionally pulled trigger	110 (23.7)
Thought gun was unloaded (not because magazine disengaged)	49 (10.6)
Thought unloaded, magazine disengaged	44 (9.5)
Gun was mistaken for a toy	36 (7.8)
Gun was dropped	24 (5.2)
Thought gun safety was engaged	15 (3.2)
Gun fired due to defect or malfunction	14 (3.0)
Gun fired while holstering	10 (2.2)
Bullet ricocheted	3 (<1.0)
Gun fired while handling safety lock	1 (<1.0)
Other mechanism of injury	75 (16.2)
**Child victim incident characteristic^§^**	
Substance use in victim’s household	10 (5.9)
Previous Child Protective Services report on victim’s household	4 (2.4)
**Total^¶^**	**464 (83.6)**

* Percentages might exceed 100% because one or more circumstances could have been known per death. Number and percentage are reported when the number of deaths is <5 because no specific circumstance identifies a single death. Denominator includes those deaths with one or more precipitating circumstances.

^†^ Includes all U.S. states and District of Columbia, with exception of Florida and Hawaii. Data for California are for violent deaths that occurred in 31 counties (Amador, Butte, Colusa, Fresno, Glenn, Humboldt, Imperial, Kings, Lake, Lassen, Los Angeles, Mendocino, Merced, Modoc, Mono, Orange, Placer, Sacramento, San Benito, San Diego, San Francisco, San Luis Obispo, San Mateo, Santa Cruz, Shasta, Siskiyou, Solano, Sonoma, Tehama, Ventura, and Yolo). Data for Texas are for violent deaths that occurred in 13 counties (Bell, Bexar, Collin, Dallas, Denton, El Paso, Fort Bend, Harris, Montgomery, Nueces, Tarrant, Travis, and Williamson).

^§^ Variables in this category apply exclusively to child victims where circumstances were known. Denominator includes decedents aged 0–17 years, n = 170. Circumstances were unknown for 20 child decedents; total unintentional firearm injury deaths in children = 190.

^¶^ Circumstances were unknown for 91 decedents; total number of unintentional firearm decedents = 555.

Known circumstances of child victim incidents were identified in 170 (89.5%) unintentional firearm injury deaths of children aged ≤18 years. Substance use problems were reported in 5.9% of child victims’ households, and 2.4% had previous Child Protective Services involvement.

### Deaths of Undetermined Intent

#### Sex, Age Group, and Race and Ethnicity

In 2021, a total of 48 NVDRS states (46 states collecting statewide data, 31 California counties, and 13 Texas counties) and the District of Columbia collected data on 5,751 incidents involving 5,806 deaths of undetermined intent (Supplementary Table 1, https://stacks.cdc.gov/view/cdc/157365). The overall rate of deaths of undetermined intent was 2.1 per 100,000 population (Supplementary Table 10, https://stacks.cdc.gov/view/cdc/157365). The rate of deaths of undetermined intent was higher among males (2.8 per 100,000 population) than females (1.4 per 100,000 population). Approximately two thirds (69.0%) of deaths of undetermined intent were among adults aged 30–64 years. The rate of deaths of undetermined intent was highest among males aged 30–34 years (4.7 per 100,000 population), followed by males aged 35–44 (4.6 per 100,000 population) and 45–54 years (4.1 per 100,000 population). The rate of deaths of undetermined intent among infants (i.e., children aged <1 year) was 2.6 per 100,000 population. Although White persons accounted for the majority (63.0%; 2.1 per 100,000 population) of deaths of undetermined intent, AI/AN persons had the highest rate (4.8 per 100,000 population). Among males, Black males (5.9 per 100,000 population) and AI/AN males (5.4 per 100,000 population) had the highest rates of deaths of undetermined intent. Among females, AI/AN females had the highest rate of deaths of undetermined intent (4.2 per 100,000 population), followed by Black females (2.1 per 100,000 population).

#### Method and Location of Injury

Poisoning was the most common method of injury in deaths of undetermined intent (65.7%), followed by a firearm (5.7%); drowning (4.0%); a blunt instrument (3.6%); a motor vehicle (2.9%); a fall (2.8%); fire or burns (2.6%); and hanging, strangulation, or suffocation (2.3%). Personal weapons, sharp instruments, intentional neglect, shaking, and other methods were each used as method of injury in <1.0% of undetermined intent deaths (Supplementary Table 10, https://stacks.cdc.gov/view/cdc/157365). Weapon type was unknown for 8.1% of undetermined intent deaths. The majority of deaths of undetermined intent occurred in a house or apartment (63.1%), followed by a street or highway (4.9%), a natural area (4.6%), or a hotel or motel (4.3%).

#### Incident Characteristics

Among all undetermined intent deaths, emergency medical services were present for a large percentage of deaths (74.2%) (Supplementary Table 10, https://stacks.cdc.gov/view/cdc/157365). Approximately half of undetermined deaths occurred at the decedent's own home (51.7%), 14.2% of decedents were suspected of alcohol use preceding the incident, 7.9% of decedents were recently released from or admitted to an institutional setting, and 4.9% of the decedents had a child present or witnessing the incident. A small proportion of deaths of undetermined intent involved decedents experiencing homelessness (5.1%) or housing instability (2.9%) at the time of death.

#### Toxicology Results of Decedent

Toxicology tests for BAC were conducted for 67.6% of decedents in deaths of undetermined intent (Supplementary Table 11, https://stacks.cdc.gov/view/cdc/157365). Among those with positive results for alcohol (37.2%), 45.0% had a BAC ≥0.08 g/dL. Tests for the following substances were conducted for the percentage of decedents indicated in parentheses: amphetamines (37.0%), antidepressants (33.2%), benzodiazepines (38.8%), cannabis (commonly referred to as marijuana; 32.7%), cocaine (46.8%), and opioids (68.9%). Among decedents tested for amphetamines, 39.2% had positive test results. Among those tested for antidepressants, 50.9% had positive results at the time of death; 38.2% of those tested for benzodiazepines had positive results, 35.2% of those tested for cannabis had positive results, and 43.2% of those tested for cocaine had positive results. Results for opioids (illegal or prescription) were positive in 77.0% of decedents tested. Carbon monoxide was tested for a substantially smaller proportion of decedents (5.6%) but was identified in 67.9% of those decedents.

#### Precipitating Circumstances

Circumstances were identified in 78.6% of deaths of undetermined intent (Supplementary Table 12, https://stacks.cdc.gov/view/cdc/157365). Among deaths of undetermined intent with known circumstances, 34.0% of decedents had a current diagnosed mental health problem at time of death. Among those with a diagnosed mental health problem the most common diagnoses were depression or dysthymia (57.7%), anxiety disorder (28.5%), and bipolar disorder (22.6%); 7.4% had depressed mood at the time of death. Substance use problems (other than alcohol; 70.1%) and alcohol problems (23.8%) were commonly reported circumstances. Among all deaths of undetermined intent, 20.7% of decedents were receiving mental health or substance use treatment at the time of death; 27.5% of decedents had a history of ever being treated for a mental health or substance use problem. A recent or impending crisis during the preceding or upcoming 2 weeks (11.7%) (types of crises listed in Supplementary Table 13, https://stacks.cdc.gov/view/cdc/157365) and physical health problems (10.5%) were other life stressors identified in deaths of undetermined intent. Among decedents, 11.4% had a history of suicidal thoughts or plans, 7.5% had a history of attempting suicide, and 4.9% had disclosed intent to die by suicide.

Circumstances were identified in 136 (52.3%) undetermined intent deaths of children and adolescents aged 0–17 years (Supplementary Table 12, https://stacks.cdc.gov/view/cdc/157365). Among child and adolescent decedents, previous Child Protective Services involvement was more frequently reported in male decedents’ household than in female decedents’ household (24.7% and 13.6%, respectively). Male decedents had a higher percentage of substance use problems in their household compared with female decedents (27.3% and 20.3%, respectively).

### Violent Deaths in Puerto Rico

For 2021, Puerto Rico collected data on 816 incidents involving 880 deaths. Homicide (n = 639) accounted for the largest proportion (72.6%) and highest rate (19.6 per 100,000 population) of these deaths, followed by suicide (n = 215; 24.4%; 7.1 per 100,000 population aged ≥10 years) (Supplementary Tables 14 and 15, https://stacks.cdc.gov/view/cdc/157365).

#### Homicides

##### Sex, Age Group, and Race and Ethnicity

In 2021, a total of 602 homicides among males and 37 homicides among females were reported in Puerto Rico (Supplementary Table 14, https://stacks.cdc.gov/view/cdc/157365). The overall homicide rate for males (39.0 per 100,000 population) was 17.7 times the rate for females (2.2 per 100,000 population). Among males, the homicide rate was 93.7 per 100,000 population among adults aged 18–29 years and 84.5 per 100,000 population among those aged 30–44 years. Most (98.7%) homicide victims were Hispanic.

##### Method and Location of Injury 

A firearm was used in a majority (91.5%) of homicides (Supplementary Table 14, https://stacks.cdc.gov/view/cdc/157365). A firearm was the most common method used in homicides of both males (93.0%) and females (67.6%); however, the firearm homicide rate for males (36.3 per 100,000 population) was 24.2 times the rate for females (1.5 per 100,000 population). Among males, a street or highway was the most common location (43.4%) of homicides, whereas a house or apartment was the most common location (40.5%) of homicides for females.

**Incident Characteristics**

Emergency medical services were present for less than one quarter of homicide victims (19.1%). A larger proportion of homicides among females than males occurred at the victim’s home (37.8% versus 7.1%, respectively). Among all homicide victims, 3.0% were suspected of alcohol use preceding the incident, 2.3% had a child who was present or witnessed the incident, and 2.0% were injured at work or while working. A small proportion of all homicide victims were experiencing homelessness (2.2%) or were recently released from an institutional setting (2.0%).

##### Victim-Suspect Relationship

The victim-suspect relationship was known for 16.3% of homicides (Supplementary Table 14, https://stacks.cdc.gov/view/cdc/157365). When the relationship was known, the suspect for male victims was most often a person known to the victim, but the exact nature of the relationship was unclear (44.2%), followed by a stranger (15.1%). Among females, the suspect was most often a current or former intimate partner (72.2%).

##### Toxicology Results of Decedent

Tests for BAC were conducted for 99.8% of homicide decedents (Supplementary Table 16, https://stacks.cdc.gov/view/cdc/157365). Among those with positive results for alcohol (32.1%), 54.1% had a BAC ≥0.08 g/dL. Tests for cocaine, cannabis (commonly referred to as marijuana), and opioids were conducted for 99.7%, 79.0%, and 99.5% of decedents, respectively. Results for cocaine, cannabis, and opioids were positive in 21.8%, 33.7%, and 9.7% of decedents tested, respectively.

##### Precipitating Circumstances

Precipitating circumstances were identified in 97.7% of homicides (Supplementary Table 17, https://stacks.cdc.gov/view/cdc/157365). Among males, more than one half (59.2%) of homicides were gang related, 30.4% involved drugs, and approximately one fifth (19.7%) involved drive-by shootings. Intimate partner violence was identified as a contributing factor in 9.0% of homicides overall; intimate partner violence precipitated 41.7% of homicides among females, compared with 7.0% of homicides among males.

#### Suicides

##### Sex, Age Group, and Race and Ethnicity

In 2021, a total of 215 suicides among persons aged ≥10 years (186 suicides among males and 29 suicides among females) were reported in Puerto Rico (Supplementary Table 15, https://stacks.cdc.gov/view/cdc/157365). The suicide rate for males was 7.2 times the rate for females (13.1 versus 1.8 per 100,000 population aged ≥10 years). Suicide rates were highest among men aged ≥65 years (16.9 per 100,000 population aged ≥10 years), followed by men aged 30–44 years (15.5 per 100,000 population aged ≥10 years). The majority (98.1%) of all suicide decedents were Hispanic.

##### Method and Location of Injury

Hanging, strangulation, or suffocation was the most commonly used method for suicide among both males (64.5%) and females (55.2%) (Supplementary Table 15, https://stacks.cdc.gov/view/cdc/157365). A firearm was used in 18.3% of suicides among males. The most common location where a suicide took place was a house or apartment, both for males (75.8%) and females (93.1%).

##### Incident Characteristics

Suicide decedents were commonly injured at their own homes (70.7%) (Supplementary Table 15, https://stacks.cdc.gov/view/cdc/157365). Among male suicide decedents, 22.6% had emergency medical services present at the incident and 6.5% were suspected of alcohol use preceding the incident.

##### Toxicology Results of Decedent

Tests for BAC were conducted for 97.7% of suicide decedents (Supplementary Table 18, https://stacks.cdc.gov/view/cdc/157365). Among those with positive results for alcohol (24.8%), 59.6% had a BAC ≥0.08 g/dL. Other than alcohol, suicide decedents were most often tested for cocaine (96.7%), cannabis (commonly referred to as marijuana; 74.4%), and opioids (97.2%). Results for cocaine, cannabis, and opioids were positive in 13.9%, 10.0%, and 6.2% of decedents tested, respectively.

##### Precipitating Circumstances

Circumstances were identified in 93.5% of suicides (Supplementary Table 19, https://stacks.cdc.gov/view/cdc/157365). Overall, a mental health problem was the most common circumstance among suicide decedents, with 50.7% having a current diagnosed mental health problem and 33.8% experiencing a depressed mood at the time of death.

Among males, 32.9% of suicide decedents had current depressed mood, and 48.0% had a current diagnosed mental health problem. Depression or dysthymia was most often the mental health diagnosis experienced by male suicide decedents with a diagnosed mental health problem (73.5%), followed by anxiety disorder (15.7%). Approximately one fourth (28.3) of male suicide decedents had a history of ever being treated for a mental health or substance use problem. Approximately one fourth (28.3%) of male suicide decedents had a history of expressing suicidal thoughts and plans, and 21.4% had a history of attempting suicide. Other precipitating circumstances for male suicide decedents included physical health problem (18.5%) and intimate partner problems (11.6%).

Among female suicide decedents, 39.3% had a current depressed mood, and 67.9% had a current diagnosed mental health problem. Depression or dysthymia was most often the mental health diagnosis experienced by female suicide decedents who had a diagnosed mental health problem (68.4%). More than half (57.1%) of female decedents had a history of ever being treated for a mental health or substance use problem, 39.3% were known to have been receiving mental health or substance use treatment at the time of death, and 42.9% had a history of attempting suicide.

## Discussion

Violent deaths affect all subgroups, regardless of sex, age, or race and ethnicity. NVDRS provides information on specific manners of death and can be used to describe characteristics of inequities experienced by populations particularly affected by fatal violence. NVDRS data also can identify common risk factors for multiple forms of violence. These details increase the knowledge base about the circumstances associated with these deaths and can assist public health authorities and their partners in developing and guiding effective, data-driven approaches to violence prevention.

The occurrence of deaths captured by NVDRS varies greatly across states, the District of Columbia, and Puerto Rico (*1*). This report summarizes data on violent deaths that occurred in 2021 in 48 NVDRS states, the District of Columbia, and Puerto Rico and describes selected characteristics. The 48 states and the District of Columbia represented 85.4% of the U.S. population (*9*) and accounted for 86.5% of violent deaths in the United States in 2021 (*1*). NVDRS contributes to measurement of the national prevention initiative Healthy People 2030 objectives related to reducing the rate of suicides, homicides, firearm-related deaths, and child abuse and neglect deaths (*13*).

Violence is preventable and reducing deaths in communities is possible with evidence-based approaches (*14*,*15*). CDC developed resources for action (i.e., technical packages) to assist communities in identifying prevention approaches that are based on the best available evidence. The resources for action describe strategies and specific programs, practices, and policies with evidence to reduce the risk for suicide, youth violence, child abuse and neglect, adverse childhood experiences, intimate partner violence, and sexual violence (*14*–*20*). Each resource for action considers the multifaceted and interactive effects of the different levels of social-ecological interrelationships, including individual, relationship, family, school, and community factors that influence violence-related outcomes. NVDRS gathers ongoing, systematic, and consistent data on deaths that can be used by prevention experts within their communities to guide planning and implementation and track outcomes of prevention strategies and approaches.

### Suicides

#### Suicide Circumstances

Approximately one third of suicide decedents had a history of suicidal thoughts or plans, and nearly one fourth had disclosed their suicidal intent. Multiple factors contribute to the risk for suicide (*21*), and the findings in this report indicate that intimate partner problems, recent or impending crises, arguments or conflicts, and physical health problems were common precipitating circumstances. The most commonly identified circumstance was mental health problems, yet approximately half of suicide decedents were not known to have a diagnosed mental health condition at the time of death. Past suicidal behavior and mental health problems are well documented as important risk factors to emphasize in suicide prevention (*15**,**22*). Less than one fourth of suicide decedents were known to be receiving treatment at the time of death, indicating a gap between those receiving treatment and those who would likely benefit from it.

Mental health problems and substance use also often co-occur among suicide victims (*23*,*24*). In this analysis, alcohol use, especially alcohol use in excess of the legal limit (i.e., BAC ≥0.08 g/dL), was frequently observed among suicide decedents who were tested for substances. Alcohol use is a strong predictor of suicidal behavior (*25*,*26*), victimization (*26*), and interpersonal violence perpetration (*27*). Intoxication can cause disinhibition, enhanced feelings of hopelessness and depression, and impaired judgment, which can lead to impulsive behaviors (*22*). In addition, positive toxicology results for opioids (illegal or prescription) were reported in approximately one fourth of suicide decedents tested for these substances. In 2017, opioid overdose was recognized as a public health emergency (*28*) after increases in opioid overdose deaths (*29*). As a result, CDC has implemented comprehensive surveillance and prevention activities through the Overdose Data to Action cooperative agreement to support state, territorial, county, and city health departments in collecting and reporting more timely and complete data on overdose morbidity and mortality and using the data to guide prevention and response efforts (*30*). In addition, CDC’s Opioid Rapid Response Program was introduced to facilitate care coordination, risk reduction, and other overdose prevention activities across Federal and state health agencies to mitigate overdose risks among patients who lose access to opioid prescribers or medications for opioid use disorder (*31*). Previous research also has suggested that chronic pain might be a contributor to suicide (*32*). The 2022 CDC Clinical Practice Guideline for Prescribing Opioids for Pain (*33*) is a tool to help clinicians and patients work together to make informed, patient-centered decisions about pain care. The guideline is intended to improve communication between clinicians and patients about the benefits and risks of pain treatments (e.g., opioid therapy for pain), improve the safety and effectiveness of pain treatment, mitigate pain, improve function and quality of life for patients with pain, and reduce the risks associated with opioid pain therapy (e.g., opioid use disorder, overdose, and death). Other important activities to address the opioid overdose epidemic include expanding naloxone availability and access to treatment with medications for opioid use disorder, enhancing public health and public safety partnerships, addressing prescription opioid misuse, and maximizing the ability of health systems to link persons who use drugs to treatment and harm-reduction services (*30*,*34*,*35*).

Another factor that might contribute to the risk for suicide is access to lethal means (e.g., firearms) among persons at risk for suicide (*15*). A firearm was the most common method used in suicides, accounting for approximately half of the deaths by suicide in this analysis. Lethal means provide limited opportunity for intervention and have high case-fatality rates (*15*). Males and older adults are more likely than females and younger adults, respectively, to use firearms as a means of suicide (*36*,*37*). This analysis found that suicide rates were highest among males and adults aged ≥75 years. Creating protective environments by reducing access to lethal means among persons at risk can be an effective strategy to prevent suicide (*15*).

#### Racial, Ethnic, and Sex-Based Inequities in Suicide Rates

Demographic variations persist in the manner of death from violence-related injuries. Suicides comprised the majority of deaths collected in NVDRS and occurred at higher rates among AI/AN and White males compared with females and other racial and ethnic groups. Men face a heightened risk of suicide compared with women which is influenced by diverse factors, including traditional norms of masculinity and reluctance to seek help for mental health problems (*38*). Specific risk factors contributing to suicide among men include rurality, access to lethal means, alcohol and drug use, relationship status, depression, and lower education levels (*39*). Further research is needed to inform the development of tailored prevention strategies among males (*38*,*40*). In addition, the findings in this report regarding suicide rates experienced by AI/AN persons, in particular, warrant attention to the contextual factors that might contribute to higher rates of suicide, such as barriers to accessing mental health care, exposure to the suicide of a friend or family member as a contributing factor to a person’s own death by suicide, relationship problems, and alcohol and substance use (*41*). Among AI/AN persons, experiences with historical trauma related to the intergenerational, collective, and cumulative effect of colonialism and ongoing inequities (e.g., discrimination, unemployment, disparaging stereotypes, microaggressions, and the resulting structural disparities) can contribute to risk for suicide (*41*,*42*). Furthermore, acknowledging the heterogeneity among persons and groups who identify as AI/AN is important (*41*,*42*).

#### The COVID-19 Pandemic and Suicide

Systemic inequities (e.g., economic, educational, housing, and employment opportunities, and structural racism) have contributed to disparities in suicide risk, and the COVID-19 pandemic could have worsened these conditions, especially in certain racial and ethnic communities (*43*,*44*). The rates of emergency department visits for mental health conditions, suicide attempts, and all drug and opioid overdoses were higher in mid-March through October 2020 during the COVID-19 pandemic compared with the same prepandemic period in 2019 (*45*). Provisional vital statistics mortality data indicate that firearm suicide rates increased approximately 11% from 2020 to 2022, with the largest increase among AI/AN persons aged 25–44 years (*44*,*46*). From 2018 to 2021, AI/AN persons experienced the highest increase in suicide rates among all racial and ethnic groups, followed by Black and Hispanic persons (*47*). Studies have highlighted the effect that the COVID-19 pandemic and mitigation measures have had on mental health, substance use, and suicide (*44*,*48*). With the experience of pandemic-related risk factors (e.g., social isolation, loss of income, and increased stress related to caregiver workload), U.S. adults reported increased symptoms of anxiety disorder, depressive disorder, new or increased substance use, and suicidal ideation (*48*). Similarly, children and adolescents experienced a significant increase in depression and anxiety symptoms during the lockdown compared with pre-lockdown rates (*49*). Contributing factors to this increase among children and adolescents might include disruptions to daily routines and schooling, changes in health care services, and housing and financial insecurity (*49*,*50*). The effects of the COVID-19 pandemic underscored the need for increased access to health services promoting social connectedness and improved diagnostic and treatment resources for mental health and substance use problems (e.g., telehealth and harm reduction services) to mitigate increases in suicidal ideation (*48*).

#### Suicide Prevention Strategies

Participating NVDRS states and jurisdictions have used VDRS data to generate reports and data visualizations to examine suicides and develop prevention efforts. For example, Maine and North Carolina have used their VDRS data to guide prevention efforts and generate reports highlighting where additional focus is needed. As part of the Maine VDRS’s work on addressing suicide among law enforcement officers, the program presented data from 2015 to 2021 to the Board of Trustees of the Maine Criminal Justice Academy, which included the Commissioner for Public Safety and the Commissioner of the Department of Corrections. Maine VDRS data and accompanying documentation from this presentation were used to mandate mental health resiliency and awareness training at the Maine Criminal Justice Academy (JA Wren, University of Maine, personal communication, October 2023). In addition, the Commissioner for the Department of Corrections has initiated plans for mandatory mental health and suicide prevention training for all corrections officers in the state. The training focuses not only on mental health issues, but also on substance use and alcohol problems (JA Wren, University of Maine, personal communication, October 2023). The North Carolina VDRS (NC-VDRS) program and data have played a considerable role in expanding activities and prevention efforts related to firearm safety and injury prevention. In 2020, an average of five persons per day died from a firearm-related death in North Carolina (>1,700 total), with more than half attributed to suicide (*51*). The NC-VDRS program offers VDRS data through their public dashboard, featuring trend data (*52*) and an annual report (*53*) that has recently been expanded to include information on firearm-related deaths. These data were used as a primary data source for partners to understand the effects of firearm-related deaths in the state. In March 2023, North Carolina’s Governor Roy Cooper issued an executive order (*54*) to create the first office of violence prevention in a southern state and the fourth in the nation to focus on reducing violence and firearm misuse in the state.

CDC’s Suicide Prevention Resource for Action: A Compilation of the Best Available Evidence identifies the following seven strategies for reducing suicide and suicidal behaviors: 1) strengthen economic supports, 2) create protective environments, 3) improve access and delivery of suicide care, 4) promote healthy connections, 5) teach coping and problem-solving skills, 6) identify and support persons at risk, and 7) lessen harms and prevent future risk. These strategies support the goals and objectives of the National Strategy for Suicide Prevention (NSSP), a comprehensive national agenda for suicide prevention (*55*), and the National Action Alliance for Suicide Prevention’s priority to strengthen community-based prevention (*56*). NVDRS is relevant to the NSSP goals of increasing timeliness and usefulness of surveillance systems related to suicide prevention and evaluating outcomes and effectiveness of suicide prevention interventions. CDC’s Suicide Prevention Resource for Action includes examples of specific approaches that communities can implement to use each strategy. The findings in this report underscore the importance of approaches outlined in the resource for action, such as social-emotional learning programs, enhanced parenting skills and family relationships, treatment for persons at risk for suicide, and treatment to prevent reattempts.

### Homicides

#### Homicides of Infants and Children

Although homicide rates for children varied across age groups, infants (i.e., children aged <1 year) experienced a higher homicide rate compared with children aged 1–14 years. Certain studies have found the highest risk for newborn and infant homicide is on the day of birth (*57*,*58*). Risk starts in infancy and continues throughout childhood, highlighting the need to prioritize strategies focused on the prevention and intervention of child abuse and neglect to reduce risk for morbidity and mortality (*19*). Child abuse and neglect often are associated with immediate physical injuries, emotional and psychological problems, involvement in risky health behaviors later in life, and a wide range of broader physical health challenges and long-term health consequences (*19*).

CDC’s Child Abuse and Neglect Prevention Resource for Action: A Compilation of the Best Available Evidence identified the following evidence-based strategies and approaches: 1) strengthening economic supports for families, 2) changing social norms to support parents and positive parenting, 3) providing quality care and education early in life, 4) enhancing parenting skills to promote healthy child development, and 5) intervening to decrease harms and prevent future risk (*19*). Child abuse and neglect are preventable, and the specific approaches described in the compilation can help create safe, stable, and nurturing relationships and environments (*59*) to prevent physical, mental, and emotional injuries as well as homicides of infants and children. The lack of safe, stable, and nurturing relationships and environments, which are essential for promoting children’s health and well-being, puts children at risk for adverse childhood experiences including violence, abuse, or death.

CDC’s Adverse Childhood Experiences Prevention Resource for Action: A Compilation of the Best Available Evidence is a comprehensive approach to preventing and mitigating the harms of adverse childhood experiences (*17*). Immediate and long-term harm of adverse childhood experiences can be lessened using multiple strategies, such as strengthening economic supports for families through work policies; promoting social norms that protect against violence and adversity via public education campaigns; ensuring a strong start for children through programs such as early childhood home visitation, quality and affordable child care, and preschool enrichment programs; connecting youths to caring adults and activities; and intervening with enhanced primary care or victim-centered services (*17*).

#### Racial and Ethnic Inequities in Homicide Rates

Racial and ethnic minority groups experience inequitable rates of violent injury and homicide, particularly among youths and young adult males (*60*). In the United States, among both males and females, AI/AN and Black persons experienced the highest rates of homicide. In Puerto Rico, the homicide rate was more than double the suicide rate, and male victims, who predominantly were Hispanic, experienced homicide rates more than double the homicide rates experienced by AI/AN and Hispanic males in the U.S. states and the District of Columbia. Racial and ethnic inequities in exposure to violence are pervasive and persistent and elimination of these inequities is an important part of a comprehensive approach to preventing violence (*60*). Racial and ethnic minority groups are disproportionately exposed to systemic inequities such as residential segregation, concentrated disadvantage, stress from experiencing racism, limited access to the best educational and employment opportunities, and other conditions that increase the risk for experiencing violence (*61*,*62*). For example, homicide rates for males in Puerto Rico have been attributed, in part, to living in communities that have been marginalized and the socioeconomic incentives of being involved in illegal means of income that are associated with high risks for violence (*63*).

Racial and ethnic minority youths often live in communities with concentrated poverty, stressed economies, residential instability, neighborhood disorganization, low community cohesion, and informal controls (*61*,*62*,*64*). All these conditions are associated with violence and violence-related injuries, and addressing the contextual factors at the structural, societal, and community levels that serve as risk factors can have broad and sustained effects in reducing racial and ethnic disparities in violence exposure (*4*,*18*,*61*,*62*,*64*). Disparity reduction strategies include policies and programs that strengthen economic and household stability, improve physical and social environments (*18*), and reduce the continuation of violence (*18*,*62*).

In conjunction with other data sources, NVDRS data can be used to help states and jurisdictions identify and address salient risk factors related to violence at the neighborhood and community levels, which can contribute to greater population-level decreases in violence through the reduction and elimination of systemic inequities (*64*). CDC’s Youth Violence Prevention Resource for Action: A Compilation of the Best Available Evidence outlines multiple programs and approaches at the community and societal levels, such as street outreach programs, environmental design activities supporting safe spaces, and policies that strengthen economic stability (*18*). For example, enhancing household financial security through tax credits (e.g., the Earned Income Tax Credit) can help families increase their income while also incentivizing work, counterbalancing the costs of child-rearing, and helping create home environments that encourage healthy childhood development (*65*). Evaluations of these programs and policies have confirmed the value of using these types of approaches to reduce the risk for violence and promote protective community environments (*14*). Evidence also suggests that these approaches and other universal policies that focus on general community improvements can have a substantial effect on decreasing racial and ethnic inequities in violence (*19*).

#### Intimate Partner Violence–Related Homicides

Homicides among males were most often precipitated by an argument or conflict or occurred during the enactment of a crime (predominately assault or homicide). In contrast, nearly half of homicides among females were intimate partner violence–related, and a current or former spouse or intimate partner was the most commonly identified suspect for female homicide victims with known suspects. Estimates from the 2016/2017 National Intimate Partner and Sexual Violence Survey indicated that approximately 111 million persons in the United States have experienced intimate partner violence (e.g., contact sexual violence, physical violence, or stalking victimization by an intimate partner) and associated adverse effects in their lifetime; furthermore, one in two females and one in four males in the United States has experienced intimate partner violence and associated adverse effects, including the experience of fear or concern for safety, at some point in their lives (*66*). Intimate partner violence–related homicides warrant further research to determine the contextual factors and characteristics of these fatal incidents and how these contextual factors might vary by various demographic characteristics.

CDC’s Intimate Partner Violence Prevention Resource for Action: A Compilation of the Best Available Evidence outlines multiple strategies for programs and policies to prevent intimate partner violence and to decrease harms (*20*). Strategies and approaches to prevent and reduce intimate partner violence might occur across different levels of social-ecological interrelationships, such as engaging men and boys as allies (*20*,*67*); disrupting developmental pathways toward intimate partner violence; creating protective school, workplace, and neighborhood environments (*20*); teaching youths about safe and healthy relationships (*20*,*68*); empowering bystanders; and strengthening economic supports for families (*20*). Prevention efforts can help change harmful gender norms that condone violence and the societal conditions that serve to maintain those norms (*20*,*69*).

#### The COVID-19 Pandemic and Homicide

Research using NVDRS data to examine the circumstances of COVID-19 pandemic era homicides is ongoing, but studies have already identified substantial changes in homicide rates. The U.S. firearm homicide rate increased by approximately 35% from 2019 to 2020 (*44*), and this trend continued in 2021 (*43*). Provisional vital statistics indicate that firearm homicide rates decreased in 2022 after a sharp increase from 2019 to 2021 (*70*). However, these rates remained substantially higher than the 2019 rate (*70*), with consistently higher rates among AI/AN and Black males (*43*,*70*). The increased social and economic stressors attributable to the COVID-19 pandemic and associated mitigation measures (e.g., job loss and disruptions in emergency services) might have exacerbated the longstanding systemic inequities that have been found to increase risk for experiencing violence, particularly among certain racial and ethnic communities (*44*,*60*,*61*). These stressors also might have contributed to increases in risk for intimate partner violence (*71*) and child abuse and neglect (*72*). Additional research is needed on the effects that the COVID-19 pandemic and mitigation measures might have had on increases in homicide rates and potential changes in the contextual factors and characteristics of these fatal incidents.

#### Homicide Prevention Strategies

NVDRS programs have used their local VDRS data to examine deaths in their states to address state public health needs. For example, in partnership with the City of Atlanta Mayor’s Office of Violence Reduction to help inform the city’s more vulnerable areas in relation to homicides, Georgia VDRS used their data to develop two public dashboards. The first dashboard provides violent death data during 2016–2021 from the entire state that includes information on trends, demographics, weapons, and circumstances surrounding the violent death, including those involving firearms (*73*). The second dashboard, including data only from the city of Atlanta, provides geographic information on health equity overlayed by health vulnerability measures including the Environmental Justice and Social-Environmental Index, comprises social vulnerability, poor built environment, lack of walkability, and high prevalence of poor mental health (*74*). These dashboards provide a critical picture of the state- and citywide geographic distribution of populations at increased risk for homicide, serving as a valuable resource to help inform violence prevention programs and policies.

The elevated homicide risk among AI/AN females has garnered national and political attention because of the underreporting of missing and murdered indigenous females in the United States (*75*–*77*). In 2016, the National Crime Information Center recorded 5,712 reports of missing AI/AN females, whereas the U.S. Department of Justice had only 116 such cases recorded in the same year (*75*). Two laws, Savanna’s Act and the Not Invisible Act, were enacted in 2020 to offer legal provisions to increase and improve data on the number of missing or murdered AI/AN persons, including AI/AN females (*78*,*79*). In 2021, President Biden signed an executive order to direct Federal agencies to work with Tribal Nations and partners to build safe and healthy tribal communities (*80*), and in 2022, he signed the Violence Against Women Act Reauthorization Act, which included new provisions to address the crisis of missing or murdered indigenous persons nationwide (*81*). Approaches that improve data collection and access (e.g., improve racial classification of records, record-keeping, and sharing of records among and by law enforcement) and promote increased and accurate media coverage have been noted as meaningful ways to address violence against AI/AN females (*75*).

### Other Manners of Death

#### Legal Intervention Deaths

NVDRS collects more complete information on legal intervention deaths than other existing data sources (*82*). The rate of legal intervention death was highest among AI/AN persons, and the rate among Black males was 2.8 times that of their White male counterparts, a finding consistent with previous studies (*83*,*84*). Racial and ethnic inequities in fatal police shootings have been examined in the literature (*85*–*87*) and have been found to be associated with factors such as increased police contact because of more traffic stops, higher presence of law enforcement in racial and ethnic minority communities, and race-based bias and perceptions of threat. More analyses are needed to increase knowledge about the magnitude and circumstances of these deaths and for developing appropriate prevention strategies and monitoring their effectiveness. Multiple strategies have been proposed and reviewed to improve policing as possible ways of decreasing legal intervention deaths (*84*,*85*,*88*–*91*). For example, studies have suggested increasing training for law enforcement to reduce potential bias in interactions with suspects, as well as training in conflict de-escalation and tactical disengagement, as approaches to reducing legal intervention deaths (*85*,*92*).

A unique strength of the NVDRS is the ability to collect data on characteristics of law enforcement officers involved in legal intervention deaths (*3*,*93*). Although not examined in this report, a previous study examining characteristics of officers involved in legal intervention deaths found associations between officer use of lethal force and characteristics such as race, age, sex, education, and previous use of force (*93*). Because of previous findings on characteristics of officers involved in legal intervention deaths and the importance of NVDRS for capturing information on legal intervention deaths, researchers have called on NVDRS to increase the completeness of demographic information on officers involved in these deaths (*84*,*93*).

#### Unintentional Firearm Injury Deaths

NVDRS also has been recognized as a reliable source of data on unintentional firearm injury deaths and for its ability to provide details about victims and shooters (*94*,*95*). In this report, approximately half of unintentional firearm injury deaths were self-inflicted; however, approximately one third were inflicted by another person. Most of these deaths occurred while the shooter was playing with the firearm, unintentionally pulling the trigger of the firearm, thinking the firearm was unloaded, or showing the firearm to others, which are concerning circumstances, particularly among children; these findings highlight the importance of secure storage practices and education about safe handling of firearms (*96*,*97*).

#### Deaths of Undetermined Intent

Poisoning was the most common method of injury in over half of deaths of undetermined intent in this report. Among those tested for each substance, a notable proportion of decedents had positive results for antidepressants, cocaine, or opioids (illegal or prescription) at the time of death. Research has demonstrated the challenges and variations in the classification of undetermined intent, particularly those due to poisoning, by coroners and medical examiners in the United States (*98*,*99*). These variations can be influenced by the definition of deaths of undetermined intent, the impact of decentralization in medicolegal practices, and the subjectivity in categorizing deaths (*99*). These findings underscore the importance of careful examination and recognition of the complexity involved in classifying of deaths of undetermined intent, emphasizing the need for consideration of this complexity in prevention efforts.

## Limitations

The findings in this report are subject to at least nine limitations. First, NVDRS data are available from only 48 states, the District of Columbia, and Puerto Rico, and therefore findings are not nationally representative. In addition, California and Texas data were from a subset of counties and are not representative of all deaths occurring in these states.

Second, although this report presents rates for NH/PI populations, Hawaii was ineligible for inclusion and the estimates in this report might not fully reflect these populations.

Third, the availability, completeness, and timeliness of data depend on partnerships among VDRS programs and local health departments, vital statistics registrars’ offices, coroners and medical examiners, and law enforcement personnel. Data sharing and communication among partners are particularly challenging when states and U.S. territories have independent county coroner or medical examiner systems (rather than a centralized coroner or medical examiner system), numerous law enforcement jurisdictions, or both. NVDRS incident data might be limited or incomplete for areas in which these data-sharing relations are not fully developed. Partnerships with local vital statistics registrars’ offices usually are more established because they are part of the public health infrastructure. As part of an active surveillance system, VDRS programs work closely with local vital registrars’ offices to identify deaths that meet the NVDRS case definition and to avoid cases being missed or inappropriately included. CDC also monitors case ascertainment and variable completeness through regular technical assistance calls, which include reviews of the internal data quality dashboard in the web-based system that is updated in real time. Overall, core variables that represent demographic characteristics (e.g., age, sex, and race and ethnicity) and manner of death were known for >99% of cases.

Fourth, toxicology data are not collected consistently across all states, the District of Columbia, and Puerto Rico or for all alcohol and drug categories. In addition, toxicology testing is not conducted for all decedents; thus, percentages of decedents with positive results for specific substances might be affected by resources available in the state or jurisdiction, infrastructure barriers, and testing practices in coroner or medical examiner offices (*100*).

Fifth, abstractors are limited to the data included in the investigative reports they receive. In addition, reports might not fully reflect all information known about an incident, particularly for homicides and legal intervention deaths, when data are less readily available until a full investigation and adjudication are completed.

Sixth, case definitions present challenges when a single death is classified differently in different documents (e.g., unintentional firearm injury death in a law enforcement report, homicide in a coroner or medical examiner record, and undetermined on the death certificate). NVDRS abstractors reconcile these discrepancies using standard NVDRS case definitions and select a single manner of death based on all source documents (*8*).

Seventh, variations in coding occur depending on the abstractor’s level of experience. For this reason, CDC provides extensive abstractor guidance and training, a coding manual to promote standardized data collection (*8*), and data validation checks. As part of their internal data quality efforts, VDRS programs are required to reabstract at least 5% of cases to examine consistency in coding and identify training needs of data abstractors.

Eighth, suicide deaths did not include children aged 5–9 years, because of the small number of suicide deaths per year in this age group. Careful interpretation is advised when comparing these counts with other studies that do not have the same age criterion (e.g., suicide data from the National Vital Statistics System) (*101*). In April 2023, the National Institute of Mental Health convened a workshop to address rising suicide rates among children and adolescents over the past two decades. A key outcome was the emphasis on raising awareness among death investigators, medical examiners, and coroners about suicides in children aged <10 years, and the importance of collecting accurate cause and manner of death information for all decedents, including children (*102*).

Finally, medical and mental health information (e.g., type of condition and whether the decedent was receiving treatment) often are not captured directly from medical records but from coroner or medical examiner records and the decedent’s family members and friends. Therefore, the completeness and accuracy of this information are limited to the knowledge of the informant.

## Future Directions

Two modules were added to NVDRS in recent years. The School-Associated Violent Death Module captures in-depth details about deaths occurring on or after January 1, 2021, on public or private K–12 school property, at a K–12 school-sponsored event, or on K-12 school–sponsored transportation. The Public Safety Officer Suicide Module covers in-depth details about incidents in which a paid, voluntary, or retired public safety officer dies by suicide on or off duty. Deaths occurring on or after January 1, 2022, are eligible for the Public Safety Officer Suicide Module. When these modules are fully onboarded across jurisdictions, they could provide critical insights into these important topics.

In accordance with CDC’s Data Modernization Initiative (*103*) and the 2021 White House Executive Order on Improving the Nation’s Cybersecurity (*104*), the NVDRS surveillance system was moved to a secure cloud-based environment in 2023. This transition has provided performance and user experience improvements to the VDRS jurisdictions. The cloud-based environment is also expected to facilitate further enhancements, such as expanded quality control measures and rapid reporting features for VDRS jurisdictions, which might help improve the fidelity, completeness, robustness, and usefulness of VDRS data.

Finally, this report summarizes data on NVDRS deaths that occurred in 2021 in 48 NVDRS states, the District of Columbia, and Puerto Rico. The goal is to include data for all 50 states in future reports.

## Conclusion

Public health surveillance is the foundation for public health practice (*99*). Monitoring the prevalence of violence-related fatal injuries, defining priorities, and informing prevention activities are essential parts of public health surveillance. In 2018, NVDRS received funding for nationwide expansion. Although not all VDRS programs’ data met the inclusion criteria for this report, all 50 states, the District of Columbia, and Puerto Rico began participating and entering data in NVDRS starting in 2019, an important step toward achieving the goal of providing nationally representative data. This expansion makes violent death information available for local communities to develop prevention efforts and allows for the system’s capacity to measure the need for and effects of violence prevention policies, programs, and practices at the national level.

## Footnotes

* Frequencies and rates of violent deaths included in this report differ slightly from the frequencies and rates of violent deaths reported by CDC’s WISQARS, which excludes nonresident deaths that occur in participating states and the District of Columbia (occurrent deaths). NVDRS tracks both resident and occurrent violent deaths in the overall data set, and the numbers in this report reflect both. VDRS programs are expected to collect information on violent deaths among their residents, wherever they occur, and fatal violent injuries occurring within their borders irrespective of the decedent’s residence status. The state of injury occurrence is responsible for collecting the information. By making this differentiation of responsibility, duplicate reporting is avoided.

† The name of the Institute of Medicine was changed to the National Academy of Medicine, effective July 1, 2015.

§ To be included in NVDRS, deaths of undetermined intent must have evidence of the possibility that the intent was purposeful, including use of a weapon or other evidence that force was used to inflict the injury. Most commonly, the coroner or medical examiner is unsure whether the death was a suicide or unintentional.

¶ In 2021, 31 California counties (Amador, Butte, Colusa, Fresno, Glenn, Humboldt, Imperial, Kings, Lake, Lassen, Los Angeles, Mendocino, Merced, Modoc, Mono, Orange, Placer, Sacramento, San Benito, San Diego, San Francisco, San Luis Obispo, San Mateo, Santa Cruz, Shasta, Siskiyou, Solano, Sonoma, Tehama, Ventura, and Yolo) contributed data to NVDRS.

** In 2021, 13 Texas counties (Bell, Bexar, Collin, Dallas, Denton, El Paso, Fort Bend, Harris, Montgomery, Nueces, Tarrant, Travis, and Williamson) contributed data to NVDRS.

†† 45 C.F.R. part 46.102(l)(2), 21 C.F.R. part 56; 42 U.S.C. Sect. 241(d); 5 U.S.C. Sect. 552a; 44 U.S.C. Sect. 3501 et seq.

§§ Drug trade is defined as the buying, selling, or passing of drugs from one person to another in exchange for goods or money.
